# Dynamics of Bacterial Community and Fermentation Quality in *Leymus chinensis* Silage Treated With Lactic Acid Bacteria and/or Water

**DOI:** 10.3389/fmicb.2021.717120

**Published:** 2021-11-03

**Authors:** Haiwen Xu, Lin Sun, Na Na, Chao Wang, Guomei Yin, Sibo Liu, Yanlin Xue

**Affiliations:** ^1^College of Foreign Languages, Inner Mongolia University of Finance and Economics, Hohhot, China; ^2^Inner Mongolia Engineering Research Center of Development and Utilization of Microbial Resources in Silage, Inner Mongolia Academy of Agriculture and Animal Husbandry Science, Hohhot, China

**Keywords:** *Leymus chinensis* silage, bacterial community, fermentation quality, lactic acid bacteria, microbial counts, moisture content

## Abstract

This study aimed to reveal the bacterial community and fermentation quality of *Leymus chinensis* silage during the fermentation process. *L. chinensis* was harvested at the heading stage, and ensiled with lactic acid bacteria (LAB, L), water (W), or a combination of both (LW) in vacuum-sealed plastic bags. As a control silage, untreated *L. chinensis* silage was also assessed. The samples were taken at 0, 5, 15, 35, and 60 days after ensiling. The bacterial community structure was assessed by plate cultivation and Illumina sequencing, and the fermentation parameters were also analyzed. Fresh *L. chinensis* contained low moisture (509 g/kg) and LAB (3.64 log colony-forming units/g fresh weight). Control silage displayed higher pH and lower lactic acid (LA) than other treatments during ensilage (*p* < 0.05); moreover, LW-treatment had lower pH from 5 to 35 days and greater LA at 5 days than L- and W-treatments (*p* < 0.05). During the fermentation process, *Lactobacillus* in L- and LW-treatments was the most dominant bacterial genus (>97%), had higher abundance than that in control silage and W-treatment (*p* < 0.05), and correlated negatively with other main genera and pH, and positively with LA and acetic acid (*p* < 0.05). Moreover, *Lactobacillus* had considerable abundance in W-treatment from 5 to 15 days (81.38–85.86%). *Enterobacteriaceae* had the most abundance among bacteria in control silage during ensiling (49.31–69.34%), and in W-treatment from 35 to 60 days (47.49–54.15%). The L-, W-, and LW-treatments displayed the aggregated bacterial community at 5 and 15 days, with W-treatment diverging from L- and LW-treatments at 35 and 60 days. Overall, the low moisture and/or insufficient LAB in fresh *L. chinensis* led to *Enterobacteriaceae* dominating bacterial community and contributing to the high pH and low LA in control silage during the fermentation process. Applying L, W, or LW contributed to *Lactobacillus* succession, LA production, and pH reduction during early stage of fermentation; moreover, treating with L and LW displayed more efficiency. *Lactobacillus* dominated the entire ensilage process in L- and LW-treatments and the early stage of fermentation in W-treatment, and contributed to the satisfactory fermentation quality of *L. chinensis* silage. The L- and LW-treatments displayed a similar pattern of bacterial succession during ensiling.

## Introduction

*Leymus chinensis* is a native cool-season perennial grass of the *Poaceae* family, and is widely distributed throughout temperate northern Asia (Zhang and Yu, 2017). It is a major forage resource for herbivores in meadow and typical steppes in Inner Mongolia, Northern China, due to its high yield, high protein content, and good palatability (Zhang et al., 2016). Ensiling is an advantageous technology for preserving *L. chinensis* that can help compensate for unreliable weather for processing hay during July and August, the principal harvest season (Xue et al., 2017). Moreover, compared with hay, silage contains higher digestible dry matter content, metabolizable energy, and crude protein, which contribute to enhanced liveweight gain (Kaiser et al., 2004).

In recent years, some studies have reported that the insufficient count of epiphytic lactic acid bacteria (LAB) in fresh *L. chinensis* contributes to high pH (5.63 and 4.31) and low lactic acid (LA, 12.5 and 17.64 g/kg DM) of silage (Zhang et al., 2015a, 2016). Additionally, Xue et al. (2017) revealed that the high dry matter (DM) content is another factor affecting fermentation quality of *L. chinensis* silage, and reported effects of location and growth stage on the nutritive value and quality of *L. chinensis* silage. Moreover, ensiling *L. chinensis* with LAB inoculants can improve the fermentation quality and aerobic stability (Tian et al., 2014; Zhang et al., 2015a,b,c; Zhang and Yu, 2017; Sun et al., 2020). Tian et al. (2014), Xue et al. (2017), and Zhang and Yu (2017) have suggested that hetero-fermentative LAB dominates the fermentation process of *L. chinensis* silage in the absence of specific inoculation, owing to the relatively low ratio of LA and acetic acid (AA) in the silage. Zhang et al. (2016) and Zhang and Yu (2017) have shown that most LAB strains isolated from *L. chinensis* silage are homofermentative. The microbial communities present in *L. chinensis* silage are not comprehensively characterized, but understanding them is critical to explaining the unique fermentation quality of its silage.

Over the past decade, the development of next-generation sequencing technologies has helped characterize the microbial communities in silage (Romero et al., 2018). This understanding is necessary to explain patterns of bacterial succession and microbial communities contributions to silage quality during the fermentation process (Wang et al., 2021). Recent studies of microbial communities have mainly focused on whole-plant corn silage (Gharechahi et al., 2017; Xu et al., 2020; Sun et al., 2021; Wang et al., 2021), alfalfa silage (Guo et al., 2018; Wang et al., 2019; Wang B. et al., 2020), sugarcane top silages (Zhang et al., 2019; Wang T. et al., 2020), and barley silage (Liu et al., 2019). To our knowledge, the microbial communities in *L. chinensis* silage have not been characterized to date.

Based on the contributions of low LAB count and high DM content in fresh *L. chinensis* to poor fermentation quality of its silage, we hypothesized that inoculating LAB and regulating moisture content at ensiling *L. chinensis* may alter bacterial community, promote beneficial bacterial successions and fermentation process, and improve fermentation quality of *L. chinensis* silage. The objective of this study was to reveal the bacterial succession pattern and fermentation quality of *L. chinensis* silage by assessing bacterial community structure and fermentation parameters during the fermentation process in *L. chinensis* silage treated with LAB and/or water.

## Materials and Methods

### Preparing Silage

*Leymus chinensis* is grown on a commercial farm (in typical steppe, 116°29′37″E, 44°13′10″N) owned by the Inner Mongolia Caodu Grassland Husbandry Co., Ltd., Xilinhot, China. The *L. chinensis* was harvested at the heading stage from four sampling sites randomly selected as replicates on July 28, 2019. The fresh forage from each site was separately chopped into 2- t o 3-cm pieces using a chaffcutter (Hongguang Industry and Trade Co., Ltd., Zhejiang, China), mixed thoroughly, and divided into four batches (3 kg for each batch) for four treatments. The compositions of the LAB inoculant were *Lactobacillus plantarum* [≥6 × 10^10^ colony-forming units (CFU)/g] and *Lactobacillus casei* (≥4 × 10^10^ CFU/g) (SYNLAC I, Viable count, ≥1.0 × 10^11^ CFU/g; Xinlaiwang Biotechnology Co., Ltd., Yangzhou, China). After mixing the LAB inoculant (2 g) with distilled water (2,000 ml), the mixture was left to rest for 2 h. The four treatments were as follows: CK (control), 2.00 ml/kg fresh weight (FW) of distilled water; L, 2.00 g/t FW of LAB inoculant, and 2.00 ml/kg FW of distilled water (2.00 ml/kg FW of the mixture); W, 100 ml/kg FW of distilled water; LW, 2.00 g/t FW of LAB inoculant and 100 ml/kg FW of distilled water (2.00 ml/kg FW of the mixture and 98 ml/kg FW of distilled water). After mixing each treatment uniformly for all samples from sampling site, approximately 500 g of each forage sample was packed into a plastic bag and sealed with a vacuum sealer. Twenty bags of silage were prepared per treatment (five bags for each treatment per sampling site). The silages were stored in the laboratory and sampled after ensiling for 0, 5, 15, 35, and 60 days. The silage sample (10 g) from each bag was placed into a labeled bag and stored at −80°C for bacterial community analysis.

### Analysis

Silage DM content was measured using a forced-air oven (BPG-9240A, Shanghai Yiheng Scientific Instrument Co., Ltd., Shanghai, China) as follows: silage was dried at 65°C for 48 h, then ground through a 1-mm screen with a mill (FS-6D; Fichi Machinery Equipment Co., Ltd., Shandong, China), and dried at 105°C until constant mass was reached. Fresh silage (25 g) and sterile water (225 ml) were homogenized for 100 s using a flap-type sterile homogenizer (JX-05, Shanghai Jingxin Industrial Development Co., Ltd., Shanghai, China) and filtered through four layers of cheesecloth to prepare silage extract (Sun et al., 2021). The pH of the silages was assessed using a pH meter (PB-10, Sartorius, Gottingen, Germany) by measuring the silage extract. Concentrations of LA, AA, propionic acid (PA), and butyric acid in the silages were assessed using high-performance liquid chromatography (DAD, 210 nm, SPD-20A, Shimadzu Co., Ltd., Kyoto, Japan) under the following conditions: detector, SPD-20A diode array detector, 210 nm; column, Shodex RS Pak KC-811, 50°C (Showa Denko K.K., Kawasaki, Japan); mobile phase, 3 mM HClO_4_, 1.0 ml/min (Wang et al., 2021). Ammonia nitrogen (NH_3_-N) and total nitrogen (TN) concentrations in the silages were assessed by a Kjeltec autoanalyzer (8400; Foss Co., Ltd., Hillerød, Denmark) using the Kjeldahl method (AOAC International, 2005). The buffering capacity (BC) was measured according to a method described by Playne and McDonald (1966).

Counts of LAB, coliforms, total aerobic bacteria, and yeast in silage were determined by culturing on Man, Rogosa, Sharpe agar, violet red bile agar, nutrient agar, and potato dextrose agar, respectively, in an incubator (LRH-70, Shanghai Yiheng Science Instruments Co., Ltd., Shanghai, China) at 30°C for 72 h (Cai, 1999).

The bacterial community in each silage sample was analyzed according to Sun et al. (2021) as follows: Bacterial DNA in the silages was extracted with an E.Z.N.A. ^®^Stool DNA Kit (D4015, Omega, Inc., United States) according to the manufacturer’s instructions. The V3–V4 region of the bacterial rRNA gene was then amplified with primers 341F (5′-CCTACGGGNGGCWGCAG-3′) and 805R (5′-GACTACHVGGGTATCTAATCC-3′) using polymerase chain reaction (PCR) with the following conditions: 98°C for 30 s followed by 32 cycles of denaturation at 98°C for 10 s, annealing at 54°C for 30 s, and extension at 72°C for 45 s, followed by a final extension at 72°C for 10 min (Logue et al., 2016). The PCR products were purified and quantified using AMPure XT beads (Beckman Coulter Genomics, Danvers, MA, United States) and Qubit (Invitrogen, United States), respectively. Purified and quantified PCR products were sequenced using an Illumina NovaSeq PE250 platform according to the manufacturer’s recommendations, provided by LC-Bio (Hangzhou Lianchuan Biotechnology Co., Ltd., Hangzhou, China). Paired-end reads were assigned to samples according to their unique barcodes. Barcode and primer sequences were then cut off. Then, paired-end reads were merged using FLASH. A total of 6,738,694 raw tags were obtained from 80 samples (84,234 raw tags per sample). The raw tags were quality filtered for high-quality clean tags using fqtrim (v0.94) under specific filtering conditions (length > 100 bp, uncertain fuzzy base numbers <5%, and quality value > 20). Low-quality and chimeric sequences (11.25% of the raw tags) were eliminated, and the rest of the sequences were filtered by Vsearch software (v2.3.4) leaving a total of 5,980,737 valid tags (74,759 valid tags per sample). The dereplication was performed using divisive amplicon denoising algorithm (DADA2). The feature table and feature sequence were obtained. A total of 4,497,859 features from 80 samples were obtained (56,223 features per sample), and the minimum sample library size was 24,344 features. Alpha and beta diversity were calculated using QIIME2. The sequences were aligned with species annotation using BLAST with a 97% similarity, and the alignment database was SILVA and NT-16S. Sequencing data were submitted to the NCBI Sequence Read Archive database (accession number: PRJNA693081). Principal component analysis (PCA) and bacterial community differences among treatments on the same day were analyzed using R 3.6.1.

### Statistical Analyses

Data on fermentation quality, microbial counts, and alpha diversity were analyzed *via* a 2 × 2 × 5 factorial design. The model includes two LAB inoculated levels, two water added levels, five sampling times, and their interactions. Interactions among LAB inoculation, water addition, and sampling time were analyzed using the PDIFF procedure of SAS (SAS System for Windows, version 9.1.3; SAS Institute Inc., Cary, NC, United States). The boxplot fermentation quality, microbial counts, and alpha diversity were built using R 3.5.0. The correlation network among the top 10 bacterial genera and a correlation heatmap of pH, LA, and AA with the top 10 bacterial genera per treatment were built using R 3.6.1.

## Results

### Fermentation Quality

**FIGURE 1:**
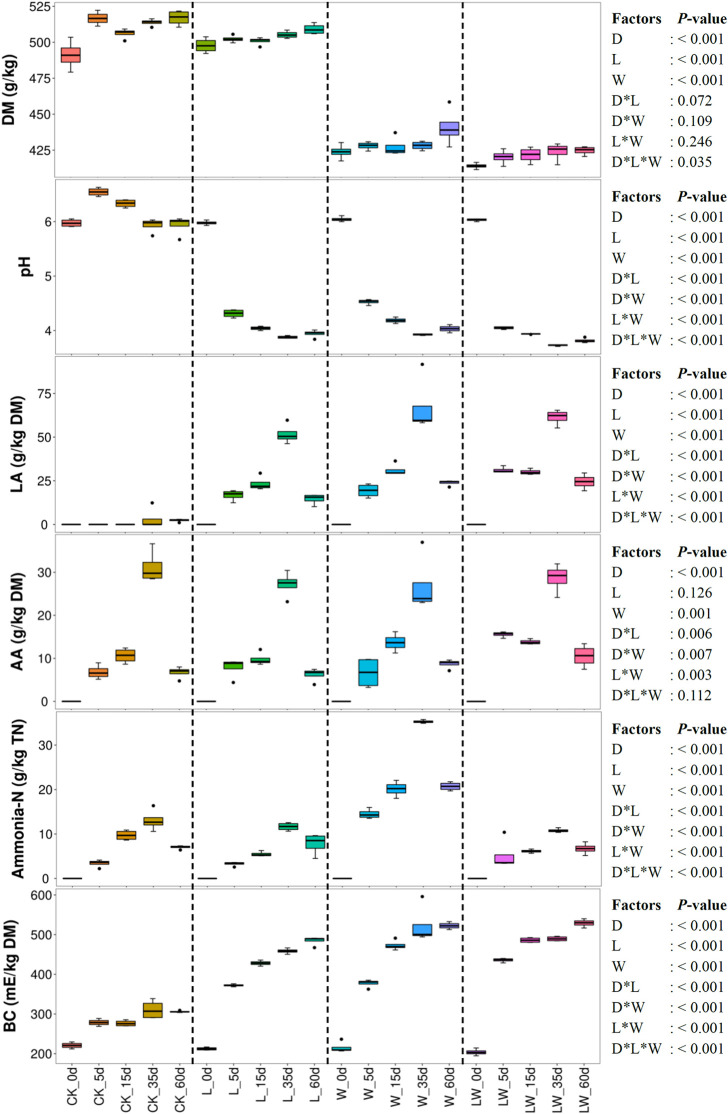
Dry matter content (DM), pH, organic acid concentrations, ammonia nitrogen/total nitrogen (Ammonia-*N*), and buffering capacity (BC) in *Leymus chinensis* silages during fermentation process (*n* = 4). CK, ensiling *L. chinensis* with 2.00 ml/kg fresh weight (FW) of distilled water; L, ensiling *L. chinensis* with 2.00 g/t FW of lactic acid bacteria (LAB) inoculant and 2.00 ml/kg FW of distilled water; W, ensiling *L. chinensis* with 100 ml/kg FW of distilled water; LW, ensiling *L. chinensis* with 2.00 g/t FW of LAB inoculant and 100.0 ml/kg FW of distilled water. LA, lactic acid; AA, acetic acid; TN, total nitrogen. D, ensiling day.

Ensiling time, LAB inoculation, and water addition had main effects and interactions on pH, LA, ammonia-*N*, and BC of silage (*p* < 0.05). The AA concentration was affected by ensiling time and water addition and was interactively influenced by ensiling time and LAB inoculation, ensiling time and water addition, and LAB inoculation and water addition (*p* < 0.05) (Figure 1). The pH in control silage increased over the first 5 days, then decreased (*p* < 0.05), but decreased in L-, W-, and LW-treatments during the ensiling process (*p* < 0.05). The LA, AA, and ammonia-*N* contents increased during the first 35 days and then decreased (*p* < 0.05) in all treatments with increasing BC (*p* < 0.05). The L-, W-, and LW-treatments displayed lower pH and greater LA and BC than control silage between 5 and 60 days (*p* < 0.05). The ammonia-*N* in W-treatment was higher than in other silages during the ensiling process (*p* < 0.05); moreover, the control silage contained higher ammonia-*N* than L- and LW-treatments at 15 days (*p* < 0.05) (Figure 1 and Supplementary Table 1). No propionic and butyric acids were detected in any of the silages.

### Microbial Counts and Diversity

**FIGURE 2:**
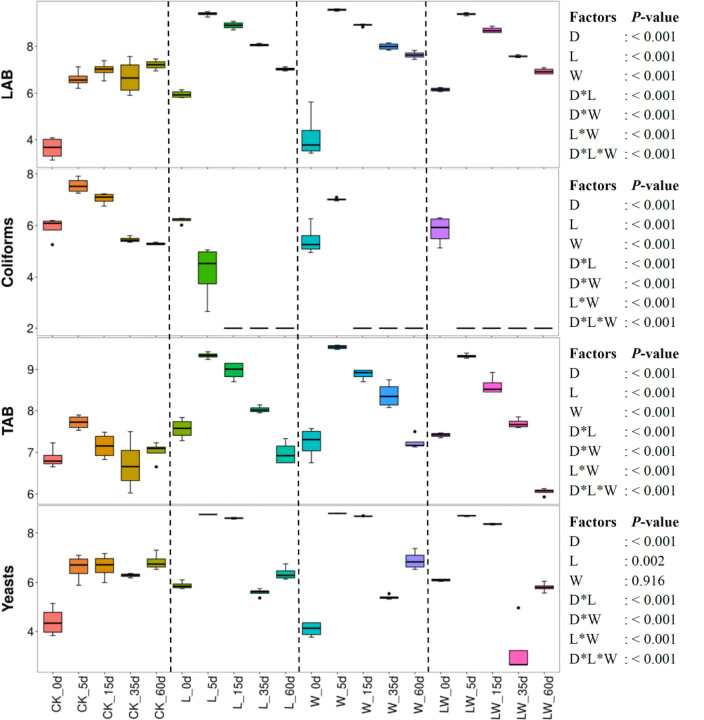
Microbial counts (log colony-forming units/g fresh weight) in *Leymus chinensis* silages during fermentation process (*n* = 4). CK, ensiling *L. chinensis* with 2.00 ml/kg fresh weight (FW) of distilled water; L, ensiling *L. chinensis* with 2.00 g/t FW of LAB inoculant and 2.00 ml/kg FW of distilled water; W, ensiling *L. chinensis* with 100 ml/kg FW of distilled water; LW, ensiling *L. chinensis* with 2.00 g/t FW of LAB inoculant and 100.0 ml/kg FW of distilled water. LAB, lactic acid bacteria; TAB, total aerobic bacteria. D, ensiling day.

Ensiling time, LAB inoculation, and water addition displayed main effects and interactions on LAB, coliforms, total aerobic bacteria, and yeast (*p* < 0.05) (Figure 2). Microbial counts increased over the first 5 days in control silage, with declining coliforms and total aerobic bacteria from 5 to 60 days (*p* < 0.05). The L-, W-, and LW-treatments displayed the increasing LAB, total aerobic bacteria, and yeast in the first 5 days, then counts in all of these categories decreased (*p* < 0.05). No coliforms in L-, W-, and LW-treatments were detected after 15, 15, and 5 days of ensiling, respectively. The L-, W-, and LW-treatments had higher LAB and total aerobic bacterial counts than control silage at 5, 15, and 35 days (*p* < 0.05), and greater yeast count at 5 and 15 days (*p* < 0.05). At 60 days, the W-treatment displayed the greatest LAB, and control silage contained the lowest total aerobic bacteria among all treatments (*p* < 0.05). At 35 and 60 days, the yeast count in LW-treatment was less than that in other treatments (*p* < 0.05) (Figure 2 and Supplementary Table 2).

**FIGURE 3:**
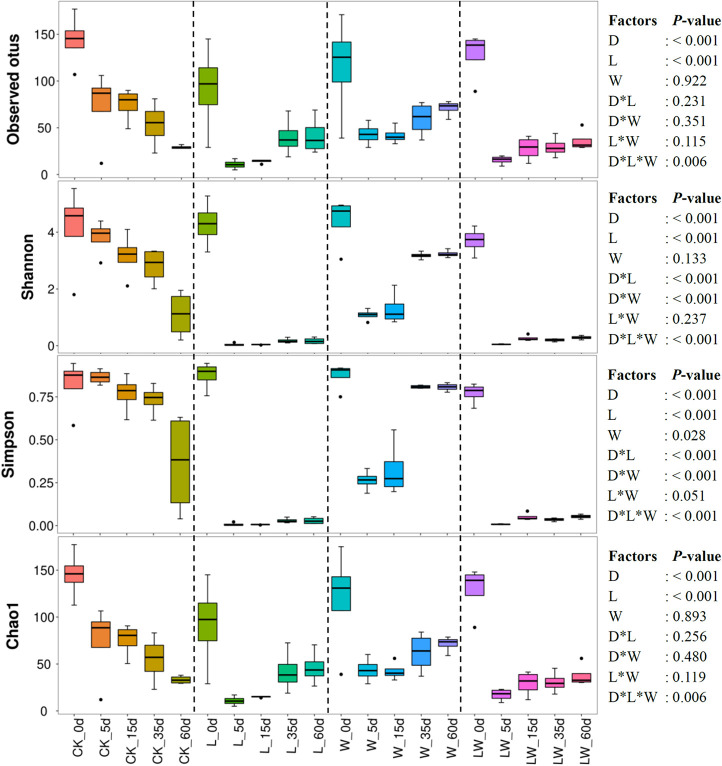
Alpha diversity of bacterial community in *Leymus chinensis* silages during fermentation process (*n* = 4). CK, ensiling *L. chinensis* with 2.00 ml/kg fresh weight (FW) of distilled water; L, ensiling *L. chinensis* with 2.00 g/t FW of lactic acid bacteria (LAB) inoculant and 2.00 ml/kg FW of distilled water; W, ensiling *L. chinensis* with 100 ml/kg FW of distilled water; LW, ensiling *L. chinensis* with 2.00 g/t FW of LAB inoculant and 100.0 ml/kg FW of distilled water. D, ensiling day.

The observed otus and the indexes of Shannon, Simpson, and Chao1 were mainly affected by ensiling time and LAB inoculation, and interactively influenced by ensiling time, LAB inoculation, and water addition (*p* < 0.05). The Shannon and Simpson indexes were also interactively affected by ensiling time and LAB inoculation, and ensiling time and water addition (*p* < 0.05). Moreover, the Shannon index was affected by water addition (*p* < 0.05) (Figure 3). The average numbers of raw reads and valid reads in silage were > 80,000 and 64,000, respectively. During the fermentation process, the observed otus and the indexes of Shannon and Chao1 decreased in all silages (*p* < 0.05). The Simpson index decreased in control silage, L-, and LW-treatments, and decreased during the first 5 days, and then increased in W-treatment (*p* < 0.05). The L-, W-, and LW-treatments had lower Shannon, Simpson, and Chao1 indexes than control silage at 5 and 10 days (*p* < 0.05). The L- and LW-treatments contained less Shannon and Simpson indexes than control and W-treatment at 35 and 60 days, and lower Chao1 index than W-treatment at 60 days (*p* < 0.05) (Figure 3 and Supplementary Table 3).

**FIGURE 4:**
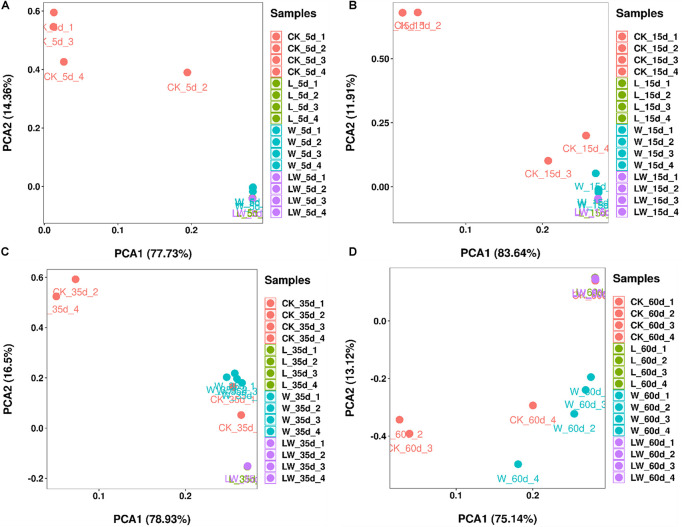
The principal component analysis of *Leymus chinensis* silage at **(A)** 5, **(B)** 15, **(C)** 35, and **(D)** 60 days after ensiling (*n* = 4). CK, ensiling *L. chinensis* with 2.00 ml/kg fresh weight (FW) of distilled water; L, ensiling *L. chinensis* with 2.00 g/t FW of LAB inoculant and 2.00 ml/kg FW of distilled water; W, ensiling *L. chinensis* with 100 ml/kg FW of distilled water; LW, ensiling *L. chinensis* with 2.00 g/t FW of LAB inoculant and 100.0 ml/kg FW of distilled water.

According to PCA analysis, the bacterial community in control silage was clearly distinct from L-, W-, and LW-treatments at 5 and 15 days (Figures 4A,B). The control and W-treatments displayed distinct bacterial communities from L- and LW-treatments at 35 and 60 days, and control silage was also distinct from W-treatment at 60 days based on PCA analysis (Figures 4C,D).

### Bacterial Community

**FIGURE 5:**
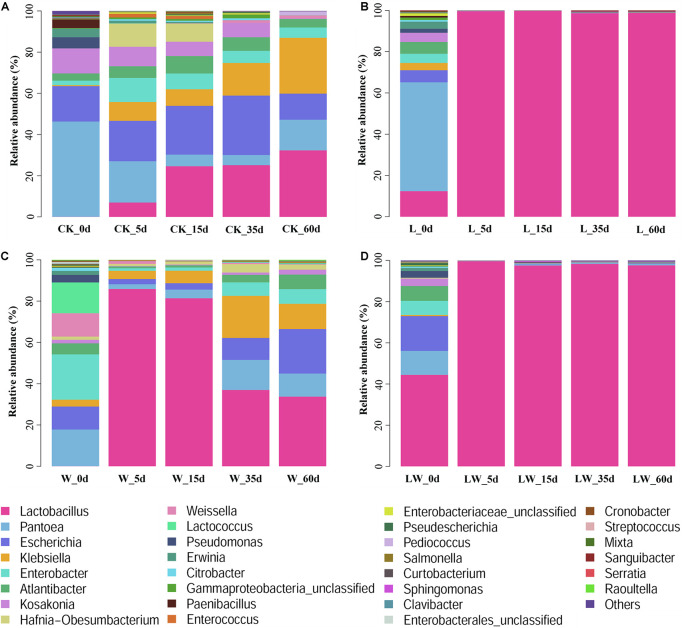
The relative abundance of bacterial community (genus level) at 0, 5, 15, 35, and 60 days after ensiling in treatments of **(A)** CK, **(B)** L, **(C)** W, and **(D)** LW of *Leymus chinensis* silage (*n* = 4). CK, ensiling *L. chinensis* with 2.00 ml/kg fresh weight (FW) of distilled water; L, ensiling *L. chinensis* with 2.00 g/t FW of lactic acid bacteria (LAB) inoculant and 2.00 ml/kg FW of distilled water; W, ensiling *L. chinensis* with 100 ml/kg FW of distilled water; LW, ensiling *L. chinensis* with 2.00 g/t FW of LAB inoculant and 100.0 ml/kg FW of distilled water.

**FIGURE 6:**
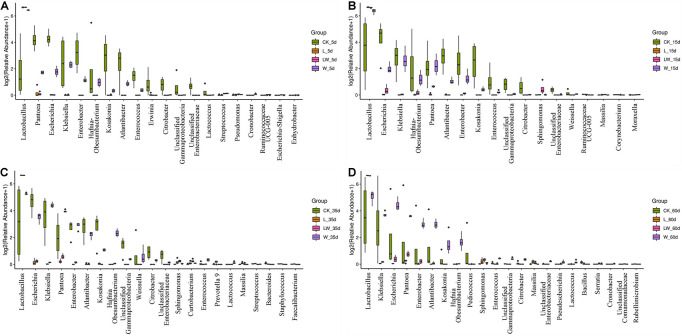
Difference in bacterial communities (genus level) among treatments of CK, L, W, and LW of *Leymus chinensis* silage at **(A)** 5, **(B)** 15, **(C)** 35, and **(D)** 60 days after ensiling (*n* = 4). CK, ensiling *L. chinensis* with 2.00 ml/kg fresh weight (FW) of distilled water; L, ensiling *L. chinensis* with 2.00 g/t FW of lactic acid bacteria (LAB) inoculant and 2.00 ml/kg FW of distilled water; W, ensiling *L. chinensis* with 100 ml/kg FW of distilled water; LW, ensiling *L. chinensis* with 2.00 g/t FW of LAB inoculant and 100.0 ml/kg FW of distilled water.

During the fermentation process, the *Lactobacillus* abundance increased slowly in control silage (from 0.07% at 0 days to 32.31% at 60 days), increased rapidly to more than 99% at 5 days then maintained a high level (>97%) in L- and LW-treatments, and increased rapidly in W-treatment to 85.86% at 5 days then decreased to 33.73% at 60 days (Figure 5). In control silage, *Pantoea* decreased over the first 35 days, then increased. The opposite trend was detected for *Escherichia*. *Enterobacter* and *Atlantibacter* increased during the first 5 and 15 days, respectively, and then decreased. *Klebsiella* increased, while *Kosakonia* decreased during the ensiling process (Figure 5A). In W-treatment, *Pantoea* decreased from 17.73% at 0 days to 2.30% at 5 days, increased to 14.57% at 35 days, then decreased to 11.17% at 60 days. *Escherichia*, *Enterobacter*, *Atlantibacter*, and *Kosakonia* decreased over the first 5 days, and then increased. *Klebsiella* increased over the first 35 days, then decreased (Figure 5C). In L- and LW-treatments, *Pantoea*, *Escherichia*, *Klebsiella*, *Enterobacter*, *Atlantibacter*, and *Kosakonia* were minor taxa from 5 to 60 days, with less than 1% abundance (Figures 5B,D). The L-, W-, and LW-treatments contained higher *Lactobacillus* and less *Pantoea* and *Escherichia* at 5 and 15 days (*p* < 0.05) relative to control silage (Figures 6A,B), and L- and LW-treatments had greater *Lactobacillus* and lower *Escherichia* and *Klebsiella* at 35 and 60 days (*p* < 0.05) (Figures 6C,D).

### Correlation Among Main Bacterial Genera

**FIGURE 7:**
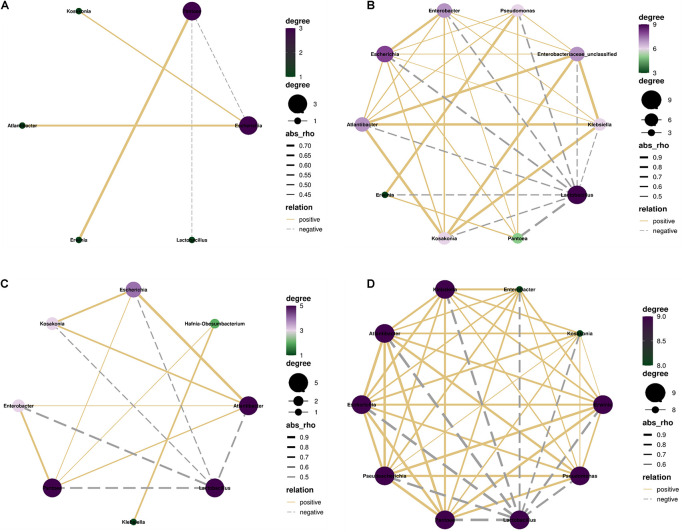
Correlation networks among main bacterial genera (top 10) in treatments of **(A)** CK, **(B)** L, **(C)** W, and **(D)** LW of *Leymus chinensis* silage (*n* = 20). CK, ensiling *L. chinensis* with 2.00 ml/kg fresh weight (FW) of distilled water; L, ensiling *L. chinensis* with 2.00 g/t FW of lactic acid bacteria (LAB) inoculant and 2.00 ml/kg FW of distilled water; W, ensiling *L. chinensis* with 100 ml/kg FW of distilled water; LW, ensiling *L. chinensis* with 2.00 g/t FW of LAB inoculant and 100.0 ml/kg FW of distilled water. *p*-value < 0.05.

In control silage, *Pantoea* correlated negatively with *Lactobacillus* and *Escherichia*, and positively with *Erwinia* (*p* < 0.05). *Escherichia* correlated positively with *Kosakonia* and *Atlantibacter* (*p* < 0.05) (Figure 7A). In W-treatment, *Lactobacillus* correlated negatively with *Pantoea*, *Escherichia*, *Atlantibacter*, *Kosakonia*, and *Enterobacter* (*p* < 0.05). *Pantoea* correlated positively with *Escherichia*, *Atlantibacter*, *Enterobacter*, and *Hafnia-Obesumbacterium* (*p* < 0.05). *Atlantibacter* correlated positively with *Escherichia*, *Kosakonia*, and *Enterobacter* (*p* < 0.05), and the positive correlations between *Klebsiella* and *Hafnia-Obesumbacterium*, and *Escherichia* and *Kosakonia* (*p* < 0.05) were detected (Figure 7C). In L- and LW-treatments, *Lactobacillus* correlated positively with the other nine bacterial genera (*p* < 0.05) (Figures 7B,D).

### Correlation Between Fermentation Quality and Main Bacterial Genera

**FIGURE 8:**
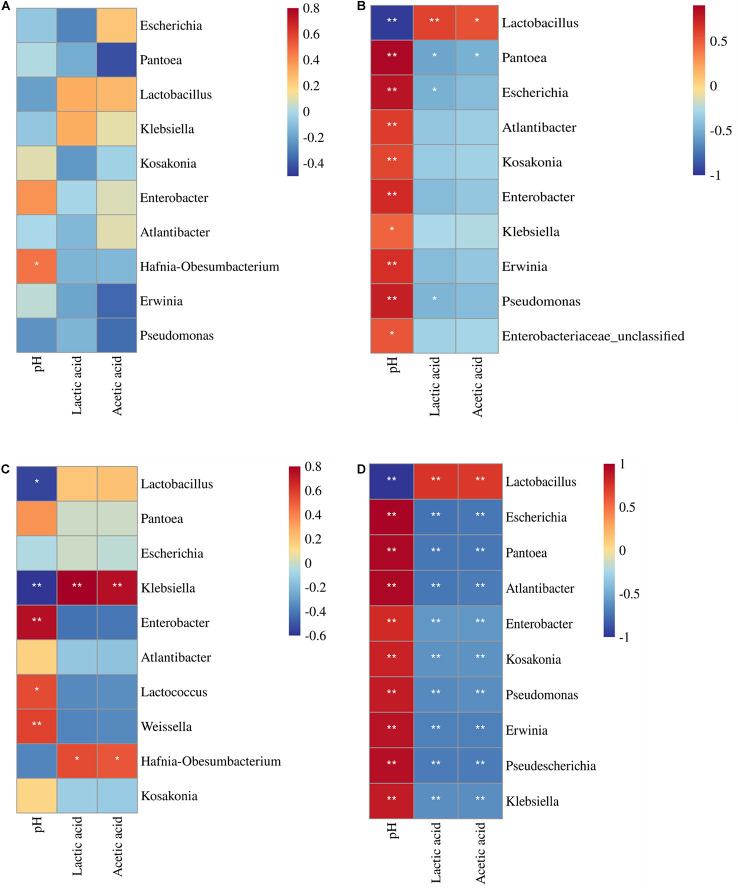
Correlation heatmap between main bacterial genera (top 10) and fermentation quality (pH, lactic acid, and acetic acid) in treatments of **(A)** CK, **(B)** L, **(C)** W, and **(D)** LW of *Leymus chinensis* silage (*n* = 20). CK, ensiling *L. chinensis* with 2.00 ml/kg fresh weight (FW) of distilled water; L, ensiling *L. chinensis* with 2.00 g/t FW of lactic acid bacteria (LAB) inoculant and 2.00 ml/kg FW of distilled water; W, ensiling *L. chinensis* with 100 ml/kg FW of distilled water; LW, ensiling *L. chinensis* with 2.00 g/t FW of LAB inoculant and 100.0 ml/kg FW of distilled water. *p < 0.05 and **p < 0.01.

In control silage, *Hafnia-Obesumbacterium* correlated positively with pH (*p* < 0.05) (Figure 8A). In L-treatment, the pH correlated negatively with *Lactobacillus*, and positively with the other nine bacterial genera (*p* < 0.05). The LA correlated positively with *Lactobacillus*, and negatively with *Pantoea*, *Escherichia*, and *Pseudomonas* (*p* < 0.05). In addition, AA correlated positively with *Lactobacillus*, and negatively with *Pantoea* (*p* < 0.05) (Figure 8B). In W-treatment, pH correlated negatively with *Lactobacillus* and *Klebsiella*, and positively with *Enterobacter*, *Lactococcus*, and *Weissella* (*p* < 0.05). Additionally, LA and AA contents correlated positively with *Klebsiella* and *Hafnia-Obesumbacterium*, respectively (*p* < 0.05) (Figure 8C). In LW-treatment, pH correlated negatively with *Lactobacillus*, and positively with the other top nine bacterial genera (*p* < 0.05). Nevertheless, LA and AA correlated positively with *Lactobacillus*, and negatively with the other top nine bacterial genera (*p* < 0.05) (Figure 8D).

## Discussion

### Characteristics of Materials

Ensiling *L. chinensis* to achieve satisfactory fermentation quality is difficult because of the low moisture content and LAB count in the material (Tian et al., 2014; Zhang et al., 2015a; Zhang and Yu, 2017). In the present study, the fresh *L. chinensis* contained low moisture content (509 g/kg) and LAB count (3.64 log CFU/g FW) (Figures 1, 2), which contributed to high pH (5.93) and coliform count (5.45 log CFU/g FW), and low LA and AA concentrations (2.32 and 6.23 g/kg DM, respectively) in control silage (Figure 1). These results were in accord with the results of Xue et al. (2017), who reported average pH, LA, and AA in *L. chinensis* silage collected from 27 sampling sites in typical steppe of 5.88, 5.14 g/kg DM, and 9.70 g/kg DM, respectively. The same study also revealed that moisture content is a primary factor affecting fermentation quality of *L. chinensis* silage, but water-soluble carbohydrate (WSC) concentration is not an important factor (Xue et al., 2017). Compared with control silage, the L-, W-, and LW-treatments had satisfactory fermentation quality, reflected by the lower pH and higher LA content, and no coliforms detected in the final silages (Figure 1 and Supplementary Tables 1, 2). Additionally, LAB were minor taxa in pre-ensiled *L. chinensis*, as reflected by the 0.20% of the bacterial community represented by LAB genera (*Lactobacillus*, *Weissella*, *Lactococcus*, *Enterococcus*, *Pediococcus*, and *Streptococcus*) (Figure 5A). These results suggest that it is necessary to inoculate LAB and modulate moisture content at ensiling *L. chinensis*.

### Characteristics of Control Silage

Previous studies deduced that heterofermentative LAB dominated the fermentation process of *L. chinensis* silage and native grass silage, due to high pH and relatively low ratio of LA to AA in final silage (Tian et al., 2014; Xue et al., 2017). In the present study, control silage had high level of pH (5.94–6.55), the LA was only detected at 35 and 60 days, and was lower than AA (Figure 1). Similar results have also been detected by Zhang et al. (2016) in *Stipa grandis* silages. However, Zhang et al. (2016) also reported that LAB strains (29) isolated from *Stipa grandis* silage were homofermentative LAB, whereas 22 LAB strains isolated from *L. chinensis* silage were homofermentative LAB, and three strains were heterofermentative LAB. Zhang and Yu (2017) found that 55% of LAB strains (97/176) isolated from *L. chinensis* silage were homofermentative LAB. Moreover, in the present study, *L. plantarum*, as the main LAB species detected in control silage, belongs to the homofermentative LAB (Supplementary Figure 1). *Escherichia*, *Klebsiella*, *Enterobacter*, *Atlantibacter*, *Kosakonia*, and *Hafnia-Obesumbacterium* were main bacterial genera in control silage (Figure 5A), and belong to the *Enterobacteriaceae.* The abundance of *Enterobacteriaceae* increased from 38.8% at 0 days to 69.3% at 5 days, then decreased to 49.3% by 60 days. *Enterobacteriaceae* was the most highly represented bacterial family in control silage from 5 to 60 days (Supplementary Figure 2). Although *Lactobacillaceae* had an increasing abundance during ensiling (from 0.07% at 0 days to 34.44% by 60 days), its abundance was lower than *Enterobacteriaceae* at each sampling time (Supplementary Figure 2). These results reveal the fact that *Enterobacteriaceae* dominates the bacterial community and regulates the fermentation process of *L. chinensis* silage without any treatment. Carvalho-Estrada et al. (2020) also reported that *Lactobacillus* was just one of main bacterial genera in high-moisture corn grain silage with *Acetobacter* as the most dominant genus because of high-level DM content (60%–70%). Sun et al. (2021) reported the increasing abundance of *Enterobacteriaceae* in whole-plant corn silage during the initial aerobic stage of fermentation.

### Characteristics of Silage Treated With Lactic Acid Bacteria and/or Water

Compared with control silage, the L-, W-, and LW-treatments at 5 and 15 days contained the aggregating bacterial communities (Figures 4A,B), and had lower pH, but higher LA content, LAB count (Figures 1, 2), and *Lactobacillus* (Figures 6A,B). Moreover, L- and LW-treatments had lower pH and higher *Lactobacillus* than W-treatment at 5 and 15 days (Figures 1, 6A,B). These results indicate that ensiling *L. chinensis* with L, W, or LW could promote *Lactobacillus* succession, and enhance early stages of fermentation (the first 15 days), and inoculating LAB without or with water had more effect than modulating moisture content alone. Previous studies have also reported that inoculating LAB at ensiling has similar effects on alfalfa silage (Guo et al., 2018), barley silages (Liu et al., 2019), oat silage (Chen et al., 2020), and whole-plant corn silage (Xu et al., 2020; Sun et al., 2021). Alfalfa silages with high moisture fermented faster with greater fermentation quality than those with low moisture (648 vs. 568, and 700 vs. 450 g/kg) (Whiter and Kung, 2001; Guo et al., 2020). In the present study, LW-treatment had the lowest pH and no coliforms detected from 5 to 60 days, and the greatest LA and AA contents at 5 days (Figure 1). Moreover, LAB inoculation and water addition exhibited interactions on pH, LA, AA, ammonia-*N*, and microbial counts (Figures 1, 2 and Supplementary Tables 1, 2). These results suggest that LW-treated *L. chinensis* silage might yield the fastest fermentation and the most vigorous microbial activity among treatments during ensilage process.

The bacterial community in W-treatment was clearly distinguishable from L- and LW-treatments at 35 and 60 days (Figures 4C,D), because the abundance of *Lactobacillus* in W-treatment was reducing (Figure 5C and Supplementary Figure 3) and lower than that in L- and LW-treatments, but greater than in the control silage (Figures 6C,D). The L- and LW-treatments had low alpha diversity during the ensilage process, which was reflected by lower Shannon, Simpson, and Chao1 indexes from 5 and 60 days (Figure 3). Moreover, for L- and LW-treatments, *Lactobacillus* was the most dominant bacterial genus, correlating negatively with the other top nine bacterial genera (Figures 7B,D). The similar correlations of top 10 bacterial genera with pH, LA, and AA were detected in L- and LW-treatments (Figures 8B,C). These results suggest that L- and LW-treated *L. chinensis* silages experienced similar bacterial succession pattern during fermentation, and the abundance of *Lactobacillus* might be the main factor influencing the bacterial succession pattern in silage.

In W-treatment, *Enterobacteriaceae* and *Erwiniaceae*, as undesirable microorganisms, had considerable abundance during ensilage process, and *Enterobacteriaceae* had the highest abundance in bacterial community at 35 and 60 days (Supplementary Figure 4). Nevertheless, *Lactobacillus* dominated the bacterial community in L- and LW-treatments from 5 to 60 days (>97%; Figures 5B,D) and in W-treatment from 5 to 15 days (>81%, Figure 5C). These results indicate that *Lactobacillus* dominates the fermentation process (from 5 to 60 days) in L- and LW-treatments, but only regulates early stage of fermentation (from 5 to 15 days) in W-treatment, and *Enterobacteriaceae* takes command of the late stage of fermentation (from 35 to 60 days) in W-treatment. Furthermore, during the fermentation process, potentially pathogenic bacteria had great abundance in control silage and W-treatment, but remained low level in L- and LW-treatments (Supplementary Figure 5). In addition, the LA content in W-treatment from 15 to 60 days did not differ from that in LW-treatment, but was higher than that in L-treatment at 15 and 60 days. The AA concentration was similar in L-, W-, and LW-treatments from 35 to 60 days (Figure 1). These results suggest that the fermentation products of the LAB inoculant used as additives in the present study could effectively inhibit undesirable microorganisms (*Enterobacteriaceae*, *Erwiniaceae*, and potentially pathogenic bacteria) during the fermentation process in *L. chinensis* silage, but the products of epiphytic LAB in *L. chinensis* exhibited poor inhibition of these organisms in control silage and W-treatment. These results abovementioned suggest that low moisture content, low LAB count, and/or metabolic characteristics of epiphytic LAB contributed to *Enterobacteriaceae* dominating the fermentation process of *L. chinensis* silage without any treatment.

### Correlation of Main Bacterial Genera With pH, Lactic Acid, and Acetic Acid

Ensiling is a complex process that requires interaction between fermentation products and microbial communities (Sun et al., 2021). Beneficial microorganisms (LAB populations) could contribute to the fermentation quality by producing a series of fermentation products (LA and AA) to reduce pH of silage (Wang T. et al., 2020). Nevertheless, the poor fermentation quality of silage may have resulted from an unsuccessful fermentation process (Guan et al., 2018), because this silage has a complex microbial community after ensiling (Carvalho-Estrada et al., 2020).

In the present study, control silage had no satisfactory fermentation quality, as reflected by higher pH (>5.9) and low LA content (2.32–3.09 g/kg DM) (Figure 1). Moreover, control silage contained higher indexes of Shannon and Simpson, and more main bacterial genera (four genera with more than 10% of average abundance) than other treatments (Figures 3, 5 and Supplementary Table 3); *Enterobacteriaceae* dominated the bacterial succession during ensilage in control silage (Supplementary Figure 2). Those abovementioned suggested that control silage had an unsuccessful fermentation process and a complex microbial community during the fermentation process. Furthermore, for control silage, pH increased from 5.98 at 0 days to 6.55 at 5 days, then decreased to 5.94 by 60 days, and LA was not detected over the first 15 days, but LAB population presented in silage during the fermentation process (Figures 1, 2, 5A). Those might be due to the fact that *Enterobacteriaceae* can thrive under anaerobic and weakly acidic conditions (McGarvey et al., 2013), and ferment WSC and LA to AA, succinic acids, ethanol, 2,3-butanediol, or other products during silage fermentation (Ni et al., 2017; Ávila and Carvalho, 2019). Moreover, *Enterobacteriaceae* in control silage correlated positively with pH and AA, and negatively with LA, although not reaching significant levels (Supplementary Figure 6). Those indicate that the LA produced by LAB in control silage from 0 to 15 days might be utilized by *Enterobacteriaceae* to AA and other products, and *Enterobacteriaceae* dominating the bacterial community resulted in the unique fermentation process.

For L- and LW-treatments, *Lactobacillus* had negative correlation with other main bacterial genera (Figure 7) and correlated negatively with pH and positively with LA and AA (Figure 8); moreover, *Lactobacillus* dominated the bacterial community during the fermentation process, with pH lower than control silage and W-treatment at 5, 15, and 60 days (Figures 1, 5 and Supplementary Table 1). Those indicate that, during the ensiling process, LAB population beat other bacteria and fermented WSC into organic acids to reduce pH rapidly in *L. chinensis* silage with L or LW. A similar result was also detected in whole-plant corn silage (Xu et al., 2020; Sun et al., 2021) and in sugarcane top silage (Wang T. et al., 2020). Furthermore, the L- and LW-treatments had less ammonia-*N* than W-treatment during the fermentation process (Figure 1 and Supplementary Table 1). Those abovementioned suggested that, in general, the satisfactory fermentation quality of silage may be attributed to *Lactobacillus* dominating the fermentation process.

For W-treatment, *Lactobacillus* had negative correlation with pH during the ensilage process (Figure 8) and correlated negatively with pH and positively with LA and AA in the first 15 days (Supplementary Figure 7A). Moreover, the most dominant bacterial genus was *Lactobacillus* at 5 and 15 days (Figure 5), and the pH reduced to 4.19 at 15 days (Figure 1). Those indicate that increasing moisture content contributed to the rapid growth of *Lactobacillus* for forming fermentation quality during early stage of fermentation in *L. chinensis* silage. However, the activity of *Lactobacillus* in W-treatment was inhibited during the late stage of the process under low pH conditions, as reflected by the reducing abundance of *Lactobacillus* and the positive correlation between *Lactobacillus* and pH from 15 to 60 days (Figure 5 and Supplementary Figure 7B). Sun et al. (2021) also detected the decreasing abundance of *Lactobacillus* from 10 to 60 days in whole-plant corn silage. Those showed that the epiphytic *Lactobacillus* of forage before ensiling has weak acid resistance.

### Ammonia-*N* of *Leymus chinensis* Silage

Proteolysis *via* the action of plant and microbial proteases is an inevitable consequence of silage fermentation (Thomas et al., 1980; Hassanat et al., 2007). The ammonia-*N* level indicates the degree of silage preservation during fermentation, because ammonia-*N*, a component of non-protein nitrogen, exhibits low utilization in the rumen (Ke et al., 2015; Xue et al., 2017; Yin et al., 2017). In the present study, ammonia-*N* remained at a low level (from 3.28 to 35.3 g/kg) during ensiling in all treatments (Figure 1). The results are in line with those reported by Xue et al. (2017), who reported that the maximum, minimum, and mean of ammonia-*N* were 97.0, 4.2, and 28.9 g/kg TN, respectively, in 27 *L. chinensis* silage samples. These results indicate that *L. chinensis* silage can be well preserved during ensilage. Moreover, the ammonia-*N* level in control silage was lower than that in W-treatment, and did not differ from L-treatment during ensiling (except at 5 days). Additionally, LW-treatment had lower ammonia-*N* and pH than W-treatment from 5 to 60 days (Supplementary Table 1). These results suggest that, for *L. chinensis* silage, DM content is a major factor contributing to preserve protein, and faster fermentation contributes to the low ammonia-*N* at high moisture level.

*Clostridia* is a minor taxon in *L. chinensis* silage during ensilage (<0.1%). However, from 5 to 60 days, the count of *Enterobacteriaceae* in control silage and W-treatment (>7 log CFU/g FW) was higher than that in L- and LW-treatments, and the *Enterobacteriaceae* count in W-treatment was the highest (Supplementary Figure 8). The ammonia-*N* also correlated positively with *Enterobacteriaceae* in *L. chinensis* silage during ensilage (Supplementary Figure 9). Moreover, *Enterobacteriaceae* is responsible for much of the ammonia-*N* formed from protein degradation and from the reduction of NO_3_ in silage (Buxton et al., 2003). Thus, *Enterobacteriaceae* activity during fermentation is a major factor influencing ammonia-*N* content in *L. chinensis* silage. Previous studies have also revealed that high DM content and inoculation with LAB at ensiling reduced ammonia-*N* in *L. chinensis* silage (Tian et al., 2014) and alfalfa silage (Whiter and Kung, 2001; Guo et al., 2020).

### Buffering Capacity of *Leymus chinensis* Silage

Fermentation of forage significantly modifies BC due to formation of lactates, acetates, and ammonia-*N*, resulting in an overall increase in BC of silage (Playne and McDonald, 1966). Therefore, *L. chinensis* silage displayed the increased BC during ensiling in the present study (Figure 1). Interestingly, the LA and BC exhibited the same trends across treatments (Figure 1). The LAB, as the main silage bacteria in W- and LW-treatments, displayed more vigorous activity than that in control silage and L-treatment, as reflected by the higher LA content at 15 and 60 days (Figure 1). The L-treatment contained more intense bacterial activity than control silage, due to higher LA content from 5 to 60 days, and greater counts of LAB and bacteria from 5 to 35 days (Figures 1, 2 and Supplementary Tables 1, 3). Moreover, the BC correlated positively with lactic acid, AA, ammonia-*N*, and counts of *Lactobacillaceae* and total bacteria (Supplementary Figures 9, 10). These results show that the intense degree of bacterial activity during ensiling contributed most strongly to the BC of *L. chinensis* silage, especially LAB activity in silage with satisfactory fermentation quality.

### Volatile Products of *Leymus chinensis* Silage

In the present study, all treatments contained lower LA and AA concentrations at 60 days than at 35 days, resulting in a slight increase in pH from 35 to 60 days (no difference in control silage and L-treatment). The ammonia-*N* also decreased from 35 to 60 days (Figure 1). These results agree with the results of Zhang et al. (2016) and Sun et al. (2020), who reported declining organic acid concentrations in *L. chinensis* silage from 30 to 45 days and from 30 to 90 days, respectively. Other previous studies also observed the decline in rehydrated corn kernel silage (Carvalho et al., 2016), smooth bromegrass silage (Niu et al., 2018), barley silage (Liu et al., 2019), whole-plant corn silages treated with inoculants (Gharechahi et al., 2017; Guan et al., 2020; Xu et al., 2020), and Napier grass silage (Tao et al., 2021). These results suggest that the declining concentration of volatile components (organic acids and ammonia-*N*) during late fermentation is a universal phenomenon in silage and needs further research.

## Conclusion

Fresh *L. chinensis* contained high DM content and low LAB count. The low moisture content and less epiphytic LAB count and/or the characteristics of epiphytic LAB in *L. chinensis* before ensiling contributed to *Enterobacteriaceae* dominating the fermentation process and resulting in high pH and low LA concentration in control silage. *Lactobacillus* dominated the entire fermentation process of L- and LW-treatments, but the early stage of fermentation in W-treatment. Ensiling *L. chinensis* with L, W, or LW could promote *Lactobacillus* succession, LA production, and pH reduction during the early stage of fermentation. The LW-treatment may yield the fastest fermentation and the most vigorous microbial activity during the ensilage process. Compared with control silage and W-treatment, treating with L and LW decreased lower pH, alpha diversity, *Enterobacteriaceae*, *Erwiniaceae*, and potentially pathogenic bacteria, and increased *Lactobacillus* during the fermentation process. The activity of *Enterobacteriaceae* during fermentation contributed to ammonia-*N* in *L. chinensis* silage and low ratio of LA to AA in control silage. The intense degree of bacterial activity during ensiling is the main factor influencing BC in *L. chinensis* silage.

## Data Availability Statement

The sequencing data were submitted to the NCBI Sequence Read Archive database (accession number: PRJNA693081).

## Author Contributions

HX, LS, and YX designed the study and wrote the manuscript. HX, LS, NN, CW, GY, and SL performed the experiments. HX and YX reviewed and edited the manuscript. LS and YX analyzed the data. YX funded and supervised the experiments. All authors reviewed the manuscript.

## Conflict of Interest

The authors declare that the research was conducted in the absence of any commercial or financial relationships that could be construed as a potential conflict of interest.

## Publisher’s Note

All claims expressed in this article are solely those of the authors and do not necessarily represent those of their affiliated organizations, or those of the publisher, the editors and the reviewers. Any product that may be evaluated in this article, or claim that may be made by its manufacturer, is not guaranteed or endorsed by the publisher.

## Supplementary Material

Supplementary Figure 1 The relative abundance of bacterial community (species level) at 0, 5, 15, 35, and 60 days after ensiling in *Leymus chinensis* silage without any treatments (*n* = 4). CK, ensiling *L. chinensis* with 2.00 mL/kg fresh weight (FW) of distilled water.

Supplementary Figure 2 The relative abundance of bacterial community (family level) at 0, 5, 15, 35, and 60 days after ensiling in *Leymus chinensis* silage without any treatments (*n* = 4). CK, ensiling *L. chinensis* with 2.00 mL/kg fresh weight (FW) of distilled water.

Supplementary Figure 3 Difference in bacterial communities (genus level) among sampling times (5, 15, 35, and 60 days after ensiling) of *Leymus chinensis* silage treated with water (*n* = 4). W, ensiling *L. chinensis* with 100 mL/kg FW of distilled water.

Supplementary Figure 4 The relative abundance of bacterial community (family level) at 0, 5, 15, 35, and 60 days after ensiling in *Leymus chinensis* silage treated with water (*n* = 4). CK, ensiling *L. chinensis* with 2.00 mL/kg fresh weight (FW) of distilled water.

Supplementary Figure 5 The relative abundance of potentially pathogenic bacteria at 0, 5, 15, 35, and 60 days after ensiling in treatments of CK, L, W, and LW of *Leymus chinensis* silage (*n* = 4). CK, ensiling *L. chinensis* with 2.00 mL/kg fresh weight (FW) of distilled water; L, ensiling *L. chinensis* with 2.00 g/t FW of LAB inoculant and 2.00 mL/kg FW of distilled water; W, ensiling *L. chinensis* with 100 mL/kg FW of distilled water; LW, ensiling *L. chinensis* with 2.00 g/t FW of LAB inoculant and 100.0 mL/kg FW of distilled water.

Supplementary Figure 6 Correlation heatmap between main bacterial families (top 10) and fermentation quality (pH, lactic acid, and acetic acid) of control silage (*n* = 20). CK (control), ensiling *L. chinensis* with 2.00 mL/kg fresh weight (FW) of distilled water. ^∗^*P* < 0.05 and ^∗∗^*P* < 0.01.

Supplementary Figure 7 Correlation heatmap between main bacterial genera (top 10) and fermentation quality (pH, lactic acid, and acetic acid) of W-treatment from 0 to 15 days **(A)** and from 15 to 60 days **(B)** (*n* = 12). W, ensiling *L. chinensis* with 100 mL/kg FW of distilled water. ^∗^*P* < 0.05 and ^∗∗^*P* < 0.01.

Supplementary Figure 8 The count of *Enterobacteriaceae* at 0, 5, 15, 35, and 60 days after ensiling in treatments of CK, L, W, and LW of *Leymus chinensis* silage (*n* = 4). CK, ensiling *L. chinensis* with 2.00 mL/kg fresh weight (FW) of distilled water; L, ensiling *L. chinensis* with 2.00 g/t FW of LAB inoculant and 2.00 mL/kg FW of distilled water; W, ensiling *L. chinensis* with 100 mL/kg FW of distilled water; LW, ensiling *L. chinensis* with 2.00 g/t FW of LAB inoculant and 100.0 mL/kg FW of distilled water. The count of *Enterobacteriaceae* = lactic acid bacteria (LAB) count ÷ relative abundance of total LAB genera × relative abundance of *Enterobacteriaceae.*

Supplementary Figure 9 Correlation heatmap of fermentation quality (pH, lactic acid, acetic acid, ammonia nitrogen/total nitrogen (NH_3_-N/TN, g/kg), and buffering capacity) with the counts of main bacterial family (top 10) and total bacteria of *Leymus chinensis* silage (*n* = 80). ^∗^*P* < 0.05 and ^∗∗^*P* < 0.01.

Supplementary Figure 10 Correlation networks among pH, lactic acid, acetic acid, ammonia nitrogen/total nitrogen (NH_3_-N/TN, g/kg), buffering capacity, and dry matter of *Leymus chinensis* silage (*n* = 80). *P*-value < 0.05.

Supplementary Table 1 Dry matter content (DM, g/kg), pH, organic acid contents (g/kg DM), ammonia nitrogen/total nitrogen (Ammonia-*N*, g/kg TN), and buffering capacity (BC, mE/kg DM) in *Leymus chinensis* silages during fermentation process (*n* = 4).

Supplementary Table 2 microbial counts (log colony forming units/g fresh weight) in *Leymus chinensis* silages during fermentation process (*n* = 4).

Supplementary Table 3 Sequencing data and alpha diversity of bacteria in *Leymus chinensis* silages during fermentation process (*n* = 4).
