# Supplementary figures and images for: Dynamics of Bacterial Community and Fermentation Quality in Leymus chinensis Silage Treated With Lactic Acid Bacteria and/or Water
Source: Front Microbiol. 2021 Nov 3;12:717120. doi: 10.3389/fmicb.2021.717120 (PMC8595406; doi:10.3389/fmicb.2021.717120)

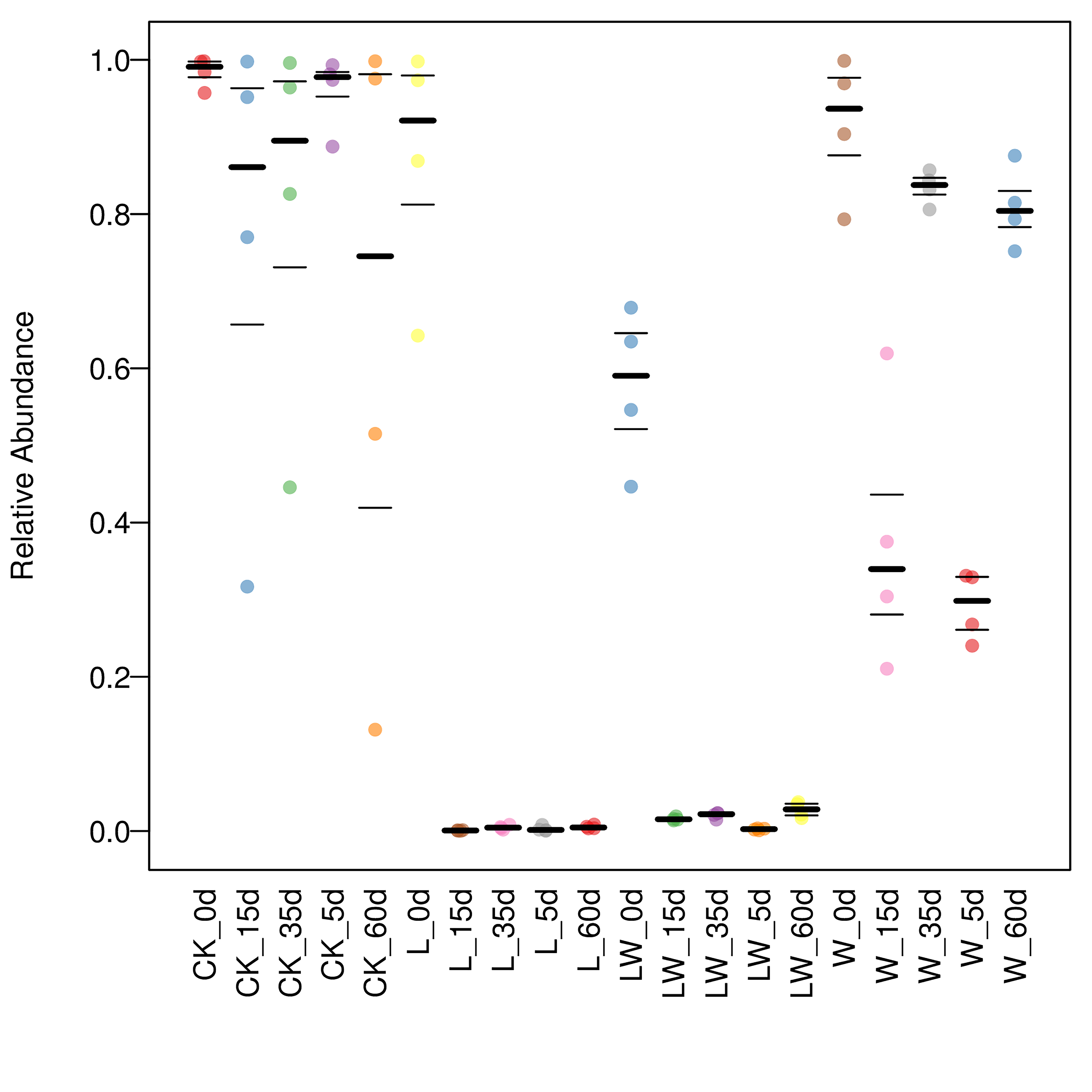

Supplement: Supplementary Figure 5 — The relative abundance of potentially pathogenic bacteria at 0, 5, 15, 35, and 60 days after ensiling in treatments of CK, L, W, and LW of Leymus chinensis silage (n = 4). CK, ensiling L. chinensis with 2.00 mL/kg fresh weight (FW) of distilled water; L, ensiling L. chinensis with 2.00 g/t FW of LAB inoculant and 2.00 mL/kg FW of distilled water; W, ensiling L. chinensis with 100 mL/kg FW of distilled water; LW, ensiling L. chinensis with 2.00 g/t FW of LAB inoculant and 100.0 mL/kg FW of distilled water. [file Image_5.PNG]

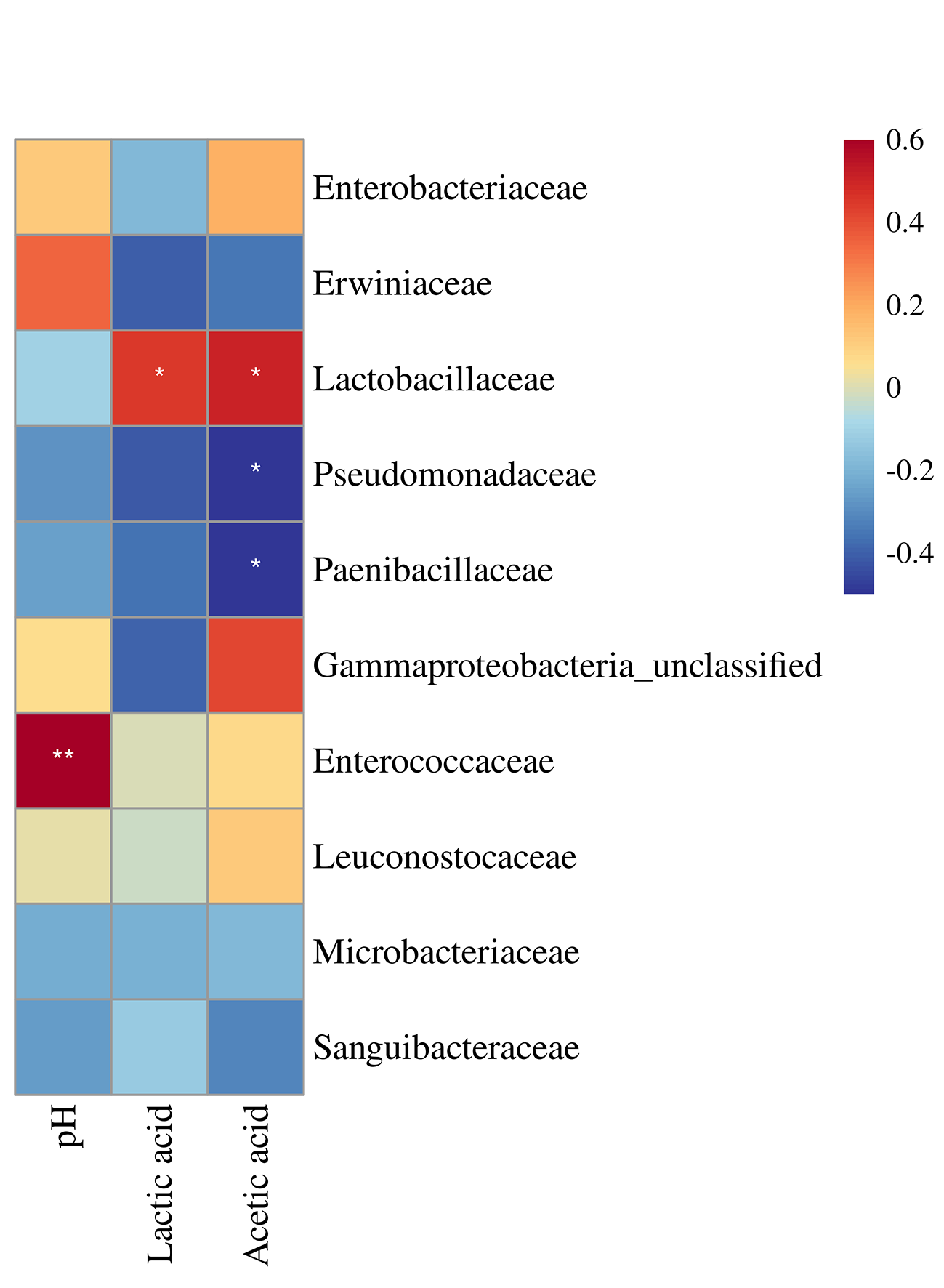

Supplement: Supplementary Figure 6 — Correlation heatmap between main bacterial families (top 10) and fermentation quality (pH, lactic acid, and acetic acid) of control silage (n = 20). CK (control), ensiling L. chinensis with 2.00 mL/kg fresh weight (FW) of distilled water. ∗P < 0.05 and ∗∗P < 0.01. [file Image_6.PNG]

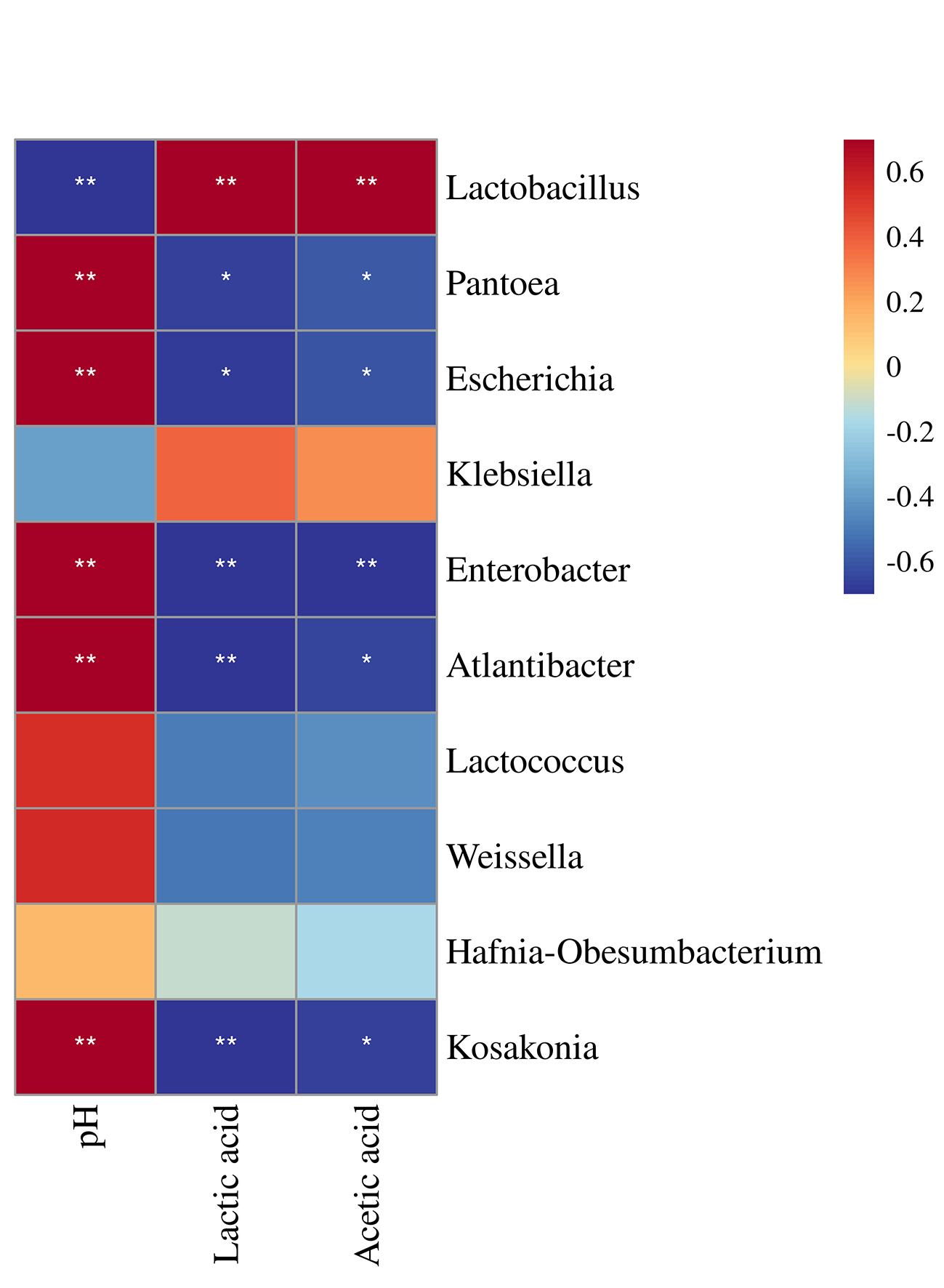

Supplement: Supplementary Figure 7 — Correlation heatmap between main bacterial genera (top 10) and fermentation quality (pH, lactic acid, and acetic acid) of W-treatment from 0 to 15 days (A) and from 15 to 60 days (B) (n = 12). W, ensiling L. chinensis with 100 mL/kg FW of distilled water. ∗P < 0.05 and ∗∗P < 0.01. [file Image_7.PNG]

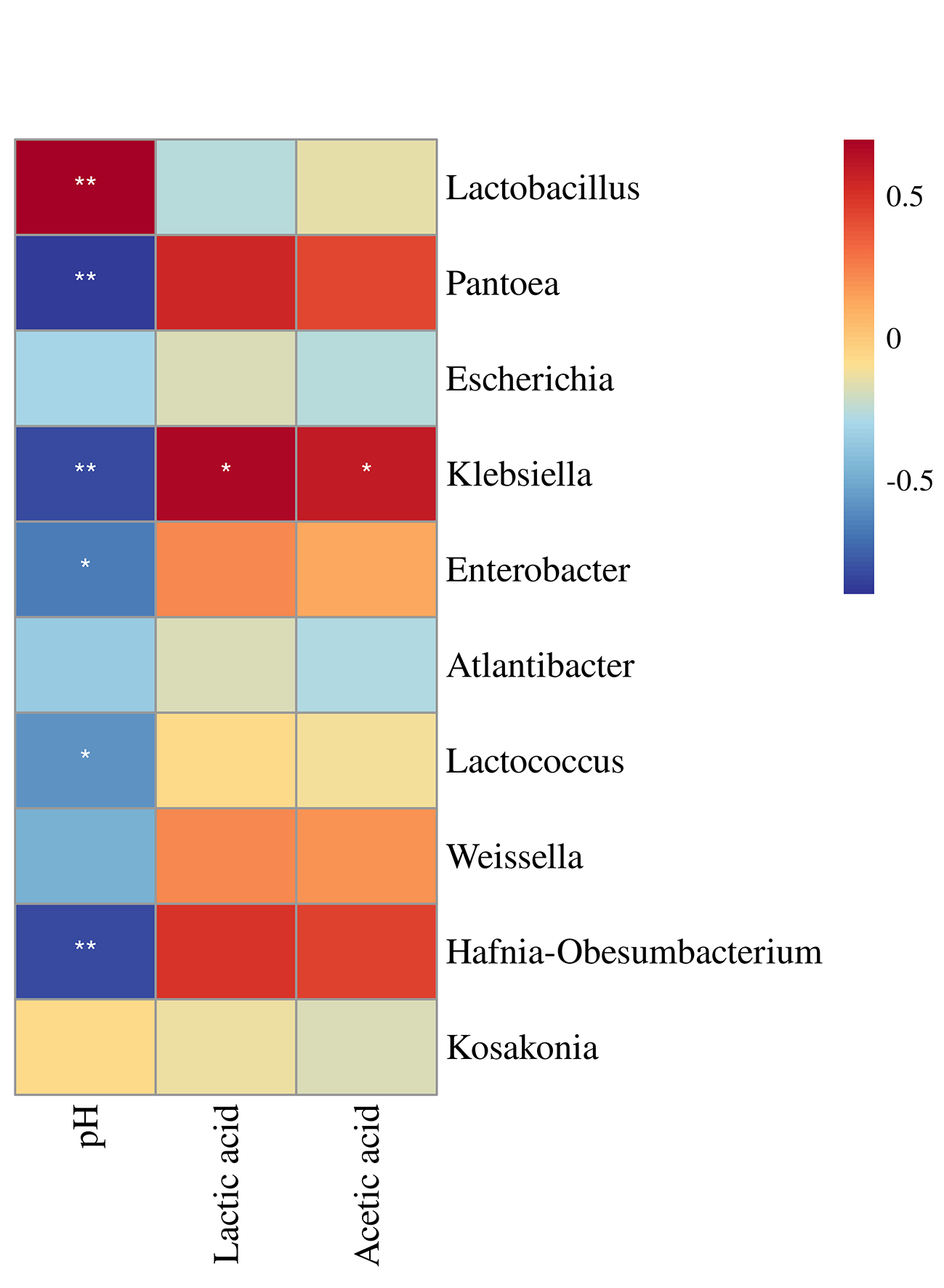

Supplement: Supplementary Figure 8 — The count of Enterobacteriaceae at 0, 5, 15, 35, and 60 days after ensiling in treatments of CK, L, W, and LW of Leymus chinensis silage (n = 4). CK, ensiling L. chinensis with 2.00 mL/kg fresh weight (FW) of distilled water; L, ensiling L. chinensis with 2.00 g/t FW of LAB inoculant and 2.00 mL/kg FW of distilled water; W, ensiling L. chinensis with 100 mL/kg FW of distilled water; LW, ensiling L. chinensis with 2.00 g/t FW of LAB inoculant and 100.0 mL/kg FW of distilled water. The count of Enterobacteriaceae = lactic acid bacteria (LAB) count ÷ relative abundance of total LAB genera × relative abundance of Enterobacteriaceae. [file Image_8.PNG]

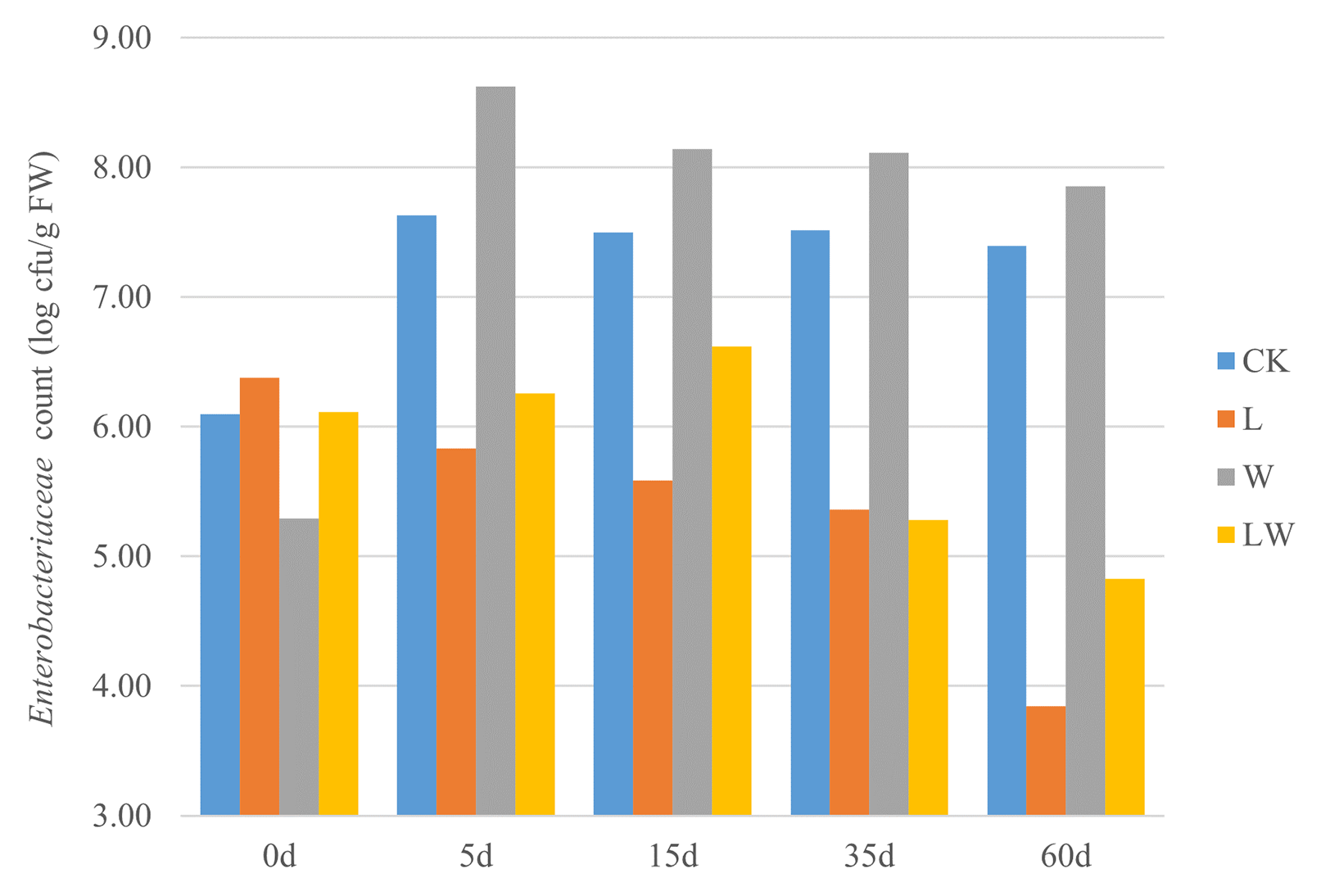

Supplement: Supplementary Figure 9 — Correlation heatmap of fermentation quality (pH, lactic acid, acetic acid, ammonia nitrogen/total nitrogen (NH3-N/TN, g/kg), and buffering capacity) with the counts of main bacterial family (top 10) and total bacteria of Leymus chinensis silage (n = 80). ∗P < 0.05 and ∗∗P < 0.01. [file Image_9.PNG]

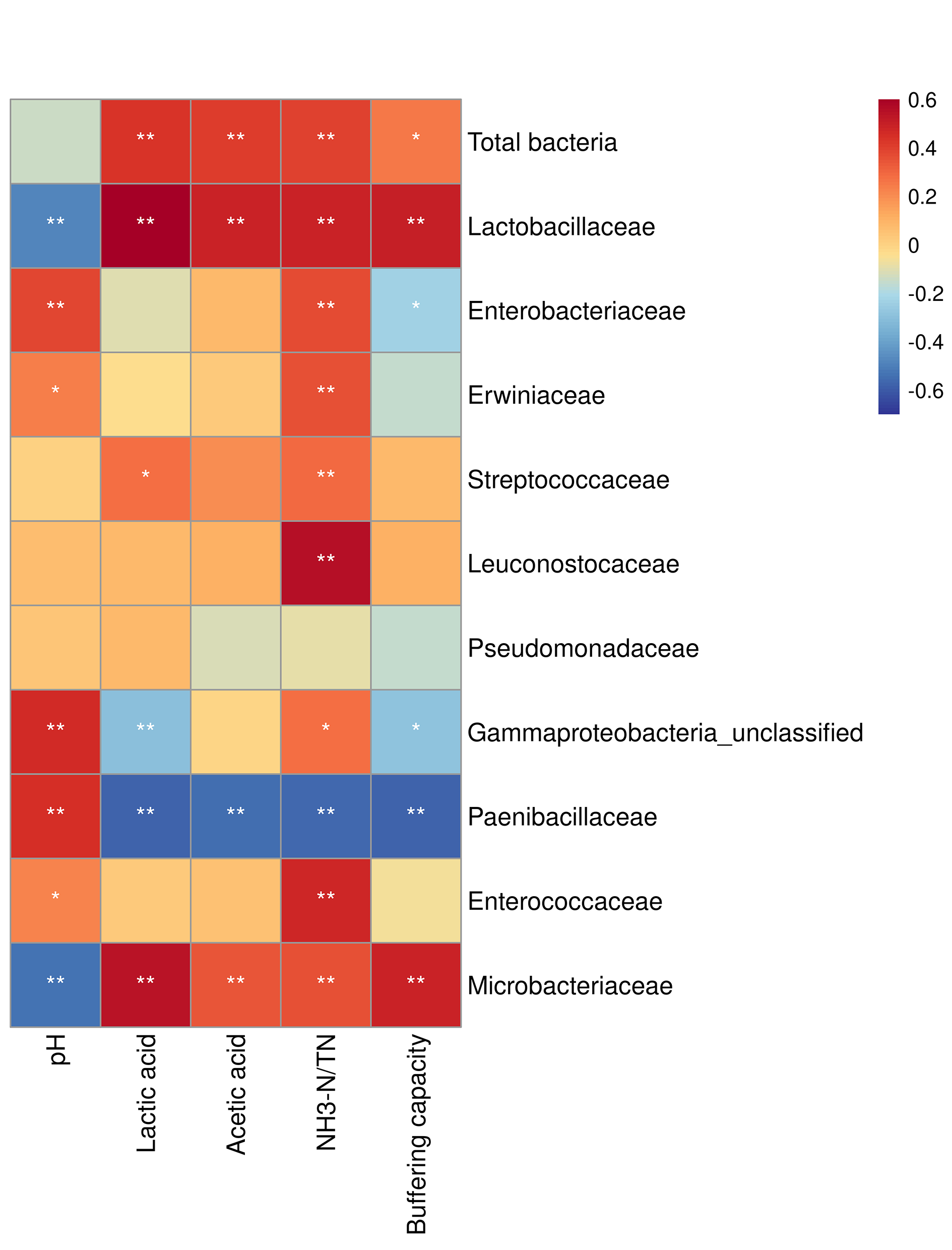

Supplement: Supplementary Figure 10 — Correlation networks among pH, lactic acid, acetic acid, ammonia nitrogen/total nitrogen (NH3-N/TN, g/kg), buffering capacity, and dry matter of Leymus chinensis silage (n = 80). P-value < 0.05. [file Image_10.PNG]

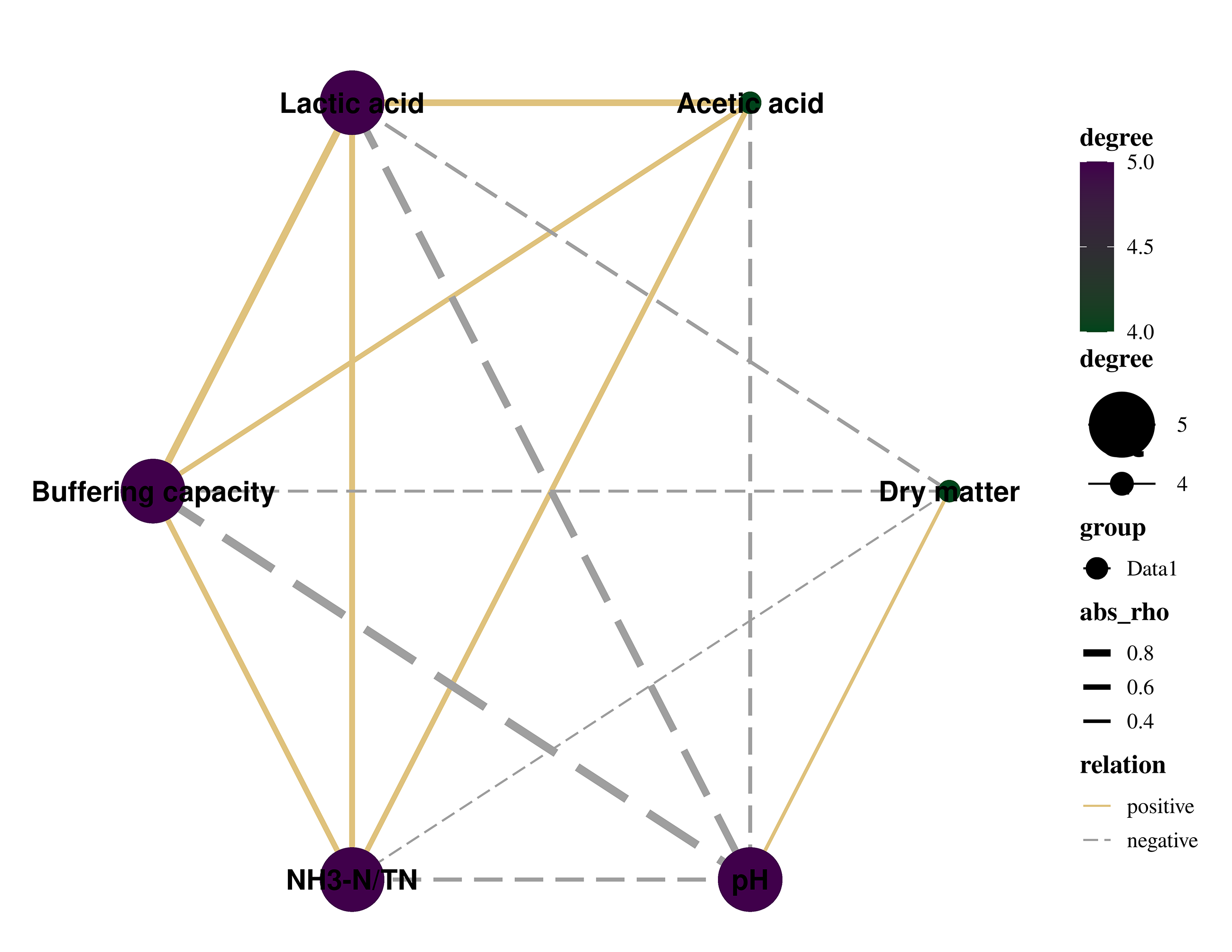

Supplement: Supplementary file 12 [file Image_11.PNG]
