# Supplementary material for: Dynamics of Bacterial Community and Fermentation Quality in Leymus chinensis Silage Treated With Lactic Acid Bacteria and/or Water
Source: Front Microbiol. 2021 Nov 3;12:717120. doi: 10.3389/fmicb.2021.717120 (PMC8595406; doi:10.3389/fmicb.2021.717120)
Supplement: Supplementary Table 1 — Dry matter content (DM, g/kg), pH, organic acid contents (g/kg DM), ammonia nitrogen/total nitrogen (Ammonia-N, g/kg TN), and buffering capacity (BC, mE/kg DM) in Leymus chinensis silages during fermentation process (n = 4). [file Table_1.DOCX]

**Table S1.** Dry matter content (DM, g/kg), pH, organic acid contents (g/kg DM), ammonia nitrogen/total nitrogen (Ammonia-N, g/kg TN), and buffering capacity (BC, mE/kg DM) in *Leymus chinensis* silages during fermentation process (n = 4).

| Items | Days | CK | L | W | LW | SEM | *P*-value | D | L | W | D*L | D*W | L*W | D*L*W |
| --- | --- | --- | --- | --- | --- | --- | --- | --- | --- | --- | --- | --- | --- | --- |
| DM | 0 | 491Ab | 498Ac | 424Bb | 414Bb | 3.22 | < 0.001 | < 0.001 | < 0.001 | < 0.001 | 0.072 | 0.109 | 0.246 | 0.035 |
|  | 5 | 517Aa | 502Bbc | 428Cab | 420Dab | 1.97 | < 0.001 |  |  |  |  |  |  |  |
|  | 15 | 506Aa | 501Abc | 427Bab | 422Bab | 2.40 | < 0.001 |  |  |  |  |  |  |  |
|  | 35 | 514Aa | 505Bab | 428Cab | 424Da | 1.89 | < 0.001 |  |  |  |  |  |  |  |
|  | 60 | 517Aa | 509Aa | 441Ba | 425Ca | 3.78 | < 0.001 |  |  |  |  |  |  |  |
|  | SEM | 3.001 | 1.76 | 3.61 | 2.27 |  |  |  |  |  |  |  |  |  |
|  | *P*-value | < 0.001 | 0.004 | 0.040 | 0.040 |  |  |  |  |  |  |  |  |  |
| pH | 0 | 5.98c | 5.98a | 6.05a | 6.03a | 0.024 | 0.133 | < 0.001 | < 0.001 | < 0.001 | < 0.001 | < 0.001 | < 0.001 | < 0.001 |
|  | 5 | 6.55Aa | 4.31Cb | 4.53Bb | 4.05Db | 0.030 | < 0.001 |  |  |  |  |  |  |  |
|  | 15 | 6.33Ab | 4.04Cc | 4.19Bc | 3.94Dc | 0.024 | < 0.001 |  |  |  |  |  |  |  |
|  | 35 | 5.93Ac | 3.88Bd | 3.93Be | 3.73Ce | 0.033 | < 0.001 |  |  |  |  |  |  |  |
|  | 60 | 5.94Ac | 3.94BCd | 4.04Bd | 3.82Cd | 0.052 | < 0.001 |  |  |  |  |  |  |  |
|  | SEM | 0.057 | 0.027 | 0.024 | 0.013 |  |  |  |  |  |  |  |  |  |
|  | *P*-value | < 0.001 | < 0.001 | < 0.001 | < 0.001 |  |  |  |  |  |  |  |  |  |
| LA | 0 | ND | ND | ND | ND | - | - | < 0.001 | < 0.001 | < 0.001 | < 0.001 | < 0.001 | < 0.001 | < 0.001 |
|  | 5 | ND | 16.7Bc | 19.4Bb | 31.2Ab | 1.31 | < 0.001 |  |  |  |  |  |  |  |
|  | 15 | ND | 23.4Bb | 31.2Ab | 30.1Ab | 1.39 | < 0.001 |  |  |  |  |  |  |  |
|  | 35 | 3.09B | 51.7Aa | 67.3Aa | 61.4Aa | 4.71 | < 0.001 |  |  |  |  |  |  |  |
|  | 60 | 2.32C | 14.6Bc | 23.8Ab | 24.5Ac | 1.40 | < 0.001 |  |  |  |  |  |  |  |
|  | SEM | 1.39 | 1.84 | 3.84 | 1.47 |  |  |  |  |  |  |  |  |  |
|  | *P*-value | 0.366 | < 0.001 | < 0.001 | < 0.001 |  |  |  |  |  |  |  |  |  |
| AA | 0 | ND | ND | ND | ND | - | - | < 0.001 | 0.126 | 0.001 | 0.006 | 0.007 | 0.003 | 0.112 |
|  | 5 | 6.81Bc | 7.82Bbc | 6.64Bc | 15.6Ab | 0.150 | < 0.001 |  |  |  |  |  |  |  |
|  | 15 | 10.6Bb | 9.81Bb | 13.7Ab | 13.8Ab | 0.794 | 0.006 |  |  |  |  |  |  |  |
|  | 35 | 31.2a | 27.2a | 26.9a | 28.7a | 2.22 | 0.536 |  |  |  |  |  |  |  |
|  | 60 | 6.72Bc | 6.20Bc | 8.72ABbc | 10.6Ac | 0.881 | 0.016 |  |  |  |  |  |  |  |
|  | SEM | 1.04 | 0.974 | 1.78 | 0.962 |  |  |  |  |  |  |  |  |  |
|  | *P*-value | < 0.001 | < 0.001 | < 0.001 | < 0.001 |  |  |  |  |  |  |  |  |  |
| Ammonia-N | 0 | ND | ND | ND | ND | - | - | < 0.001 | < 0.001 | < 0.001 | < 0.001 | < 0.001 | < 0.001 | < 0.001 |
|  | 5 | 3.40Bd | 3.28Bd | 14.5Ac | 5.22Bb | 0.9369 | < 0.001 |  |  |  |  |  |  |  |
|  | 15 | 9.72Bb | 5.49Cc | 20.1Ab | 6.14Cb | 0.5477 | < 0.001 |  |  |  |  |  |  |  |
|  | 35 | 13.1Ba | 11.6Ba | 35.3Aa | 10.8Ba | 0.6661 | < 0.001 |  |  |  |  |  |  |  |
|  | 60 | 7.02Bc | 7.80Bb | 20.7Ab | 6.72Bb | 0.7278 | < 0.001 |  |  |  |  |  |  |  |
|  | SEM | 0.633 | 0.598 | 0.516 | 0.883 |  |  |  |  |  |  |  |  |  |
|  | *P*-value | < 0.001 | < 0.001 | < 0.001 | < 0.001 |  |  |  |  |  |  |  |  |  |
| BC | 0 | 221c | 212e | 216e | 204d | 4.60 | 0.129 | < 0.001 | < 0.001 | < 0.001 | < 0.001 | < 0.001 | < 0.001 | < 0.001 |
|  | 5 | 279Cb | 372Bd | 377Bd | 436Ac | 3.59 | < 0.001 |  |  |  |  |  |  |  |
|  | 15 | 277Cb | 428Bc | 473Ac | 486Ab | 4.38 | < 0.001 |  |  |  |  |  |  |  |
|  | 35 | 311Ca | 459Bb | 498Ab | 490ABb | 6.47 | < 0.001 |  |  |  |  |  |  |  |
|  | 60 | 306Ca | 484Ba | 523Aa | 529Aa | 4.38 | < 0.001 |  |  |  |  |  |  |  |
|  | SEM | 6.24 | 3.46 | 5.19 | 3.69 |  |  |  |  |  |  |  |  |  |
|  | *P*-value | < 0.001 | < 0.001 | < 0.001 | < 0.001 |  |  |  |  |  |  |  |  |  |

CK, ensiling *L. chinensis* with 2.00 mL/kg fresh weight (FW) of distilled water; L, ensiling *L. chinensis* with 2.00 g/t FW of LAB inoculant and 2.00 mL/kg FW of distilled water; W, ensiling *L. chinensis* with 100 mL/kg FW of distilled water; LW, ensiling *L. chinensis* with 2.00 g/t FW of LAB inoculant and 100.0 mL/kg FW of distilled water. SEM, standard error of the mean. D, days after ensiling. Values with different uppercase letters (A, B, C, and D) indicate significant differences among treatments on the same day (*P* < 0.05). Values with different lowercase letters (a, b, ……, e) indicate significant differences among days after ensiling for the same treatment (*P* < 0.05). LA, lactic acid; AA, acetic acid. ND, not detected.

**Table S2.** microbial counts (log colony forming units/g fresh weight) in *Leymus chinensis* silages during fermentation process (n = 4).

| Items | Days | CK | L | W | LW^4^ | SEM | *P*-value | D | L | W | D*L | D*W | L*W | D*L*W |
| --- | --- | --- | --- | --- | --- | --- | --- | --- | --- | --- | --- | --- | --- | --- |
| LAB | 0 | 3.64Bb | 5.95Ae | 4.15Bc | 6.16Ae | 0.291 | < 0.001 | < 0.001 | < 0.001 | < 0.001 | < 0.001 | < 0.001 | < 0.001 | < 0.001 |
|  | 5 | 6.62Ba | 9.39Aa | 9.56Aa | 9.38Aa | 0.099 | < 0.001 |  |  |  |  |  |  |  |
|  | 15 | 6.99Ba | 8.90Ab | 8.91Aa | 8.70Ab | 0.103 | < 0.001 |  |  |  |  |  |  |  |
|  | 35 | 6.70Ba | 8.06Ac | 8.00Ab | 7.58Ac | 0.196 | 0.001 |  |  |  |  |  |  |  |
|  | 60 | 7.21Ba | 7.04Bd | 7.64Ab | 6.94Bd | 0.078 | < 0.001 |  |  |  |  |  |  |  |
|  | SEM | 0.238 | 0.059 | 0.230 | 0.046 |  |  |  |  |  |  |  |  |  |
|  | *P*-value | < 0.001 | < 0.001 | < 0.001 | < 0.001 |  |  |  |  |  |  |  |  |  |
| Coliforms | 0 | 5.90c | 6.20a | 5.43b | 5.82a | 0.2309 | 0.189 | < 0.001 | < 0.001 | < 0.001 | < 0.001 | < 0.001 | < 0.001 | < 0.001 |
|  | 5 | 7.55Aa | 4.19Bb | 7.01Aa | ND | 0.288 | < 0.001 |  |  |  |  |  |  |  |
|  | 15 | 7.05Ab | ND | ND | ND | 0.055 | < 0.001 |  |  |  |  |  |  |  |
|  | 35 | 5.45Ad | ND | ND | ND | 0.028 | < 0.001 |  |  |  |  |  |  |  |
|  | 60 | 5.29Ad | ND | ND | ND | 0.011 | < 0.001 |  |  |  |  |  |  |  |
|  | SEM | 0.131 | 0.249 | 0.130 | 0.124 |  |  |  |  |  |  |  |  |  |
|  | *P*-value | < 0.001 | < 0.001 | < 0.001 | < 0.001 |  |  |  |  |  |  |  |  |  |
| Total aerobic bacteria | 0 | 6.86Bb | 7.57Ad | 7.23ABd | 7.42Ad | 0.129 | 0.013 | < 0.001 | < 0.001 | < 0.001 | < 0.001 | < 0.001 | < 0.001 | < 0.001 |
|  | 5 | 7.72Ca | 9.33Ba | 9.54Aa | 9.32Ba | 0.050 | < 0.001 |  |  |  |  |  |  |  |
|  | 15 | 7.15Bb | 8.97Ab | 8.88Ab | 8.60Ab | 0.116 | < 0.001 |  |  |  |  |  |  |  |
|  | 35 | 6.71Bb | 8.03Ac | 8.38Ac | 7.69Ac | 0.181 | < 0.001 |  |  |  |  |  |  |  |
|  | 60 | 7.02Ab | 6.98Ae | 7.24Ad | 6.06Be | 0.106 | < 0.001 |  |  |  |  |  |  |  |
|  | SEM | 0.1820 | 0.101 | 0.120 | 0.062 |  |  |  |  |  |  |  |  |  |
|  | *P*-value | 0.013 | < 0.001 | < 0.001 | < 0.001 |  |  |  |  |  |  |  |  |  |
| Yeasts | 0 | 4.41Bb | 5.88Ac | 4.10Bd | 6.09Ab | 0.172 | < 0.001 | < 0.001 | 0.002 | 0.916 | < 0.001 | < 0.001 | < 0.001 | < 0.001 |
|  | 5 | 6.60Ba | 8.75Aa | 8.80Aa | 8.70Aa | 0.133 | < 0.001 |  |  |  |  |  |  |  |
|  | 15 | 6.65Ba | 8.60Aa | 8.68Aa | 8.36Aa | 0.127 | < 0.001 |  |  |  |  |  |  |  |
|  | 35 | 6.28Aa | 5.58Ad | 5.38Ac | 3.23Bc | 0.292 | < 0.001 |  |  |  |  |  |  |  |
|  | 60 | 6.83Aa | 6.36Ab | 6.89Ab | 5.80Bb | 0.153 | 0.0009 |  |  |  |  |  |  |  |
|  | SEM | 0.226 | 0.081 | 0.110 | 0.261 |  |  |  |  |  |  |  |  |  |
|  | *P*-value | < 0.001 | < 0.001 | < 0.001 | < 0.001 |  |  |  |  |  |  |  |  |  |

CK, ensiling *L. chinensis* with 2.00 mL/kg fresh weight (FW) of distilled water; L, ensiling *L. chinensis* with 2.00 g/t FW of LAB inoculant and 2.00 mL/kg FW of distilled water; W, ensiling *L. chinensis* with 100 mL/kg FW of distilled water; LW, ensiling *L. chinensis* with 2.00 g/t FW of LAB inoculant and 100.0 mL/kg FW of distilled water. SEM, standard error of the mean. D, days after ensiling. Values with different uppercase letters (A, B, C, and D) indicate significant differences among treatments on the same day (*P* < 0.05). Values with different lowercase letters (a, b, ……, e) indicate significant differences among days after ensiling for the same treatment (*P* < 0.05). LAB, lactic acid bacteria. ND, not detected.

**Table S3.** Sequencing data and alpha diversity of bacteria in *Leymus chinensis* silages during fermentation process (n = 4).

| Items | Days | CK | L | W | LW | SEM | *P*-value | D | L | W | D*L | D*W | L*W | D*L*W |
| --- | --- | --- | --- | --- | --- | --- | --- | --- | --- | --- | --- | --- | --- | --- |
| Raw tags | 0 | 84360 | 80529 | 83337 | 83563 | 2535 | 0.732 | 0.245 | 0.254 | 0.972 | 0.684 | 0.313 | 0.214 | 0.590 |
|  | 5 | 90439 | 83954 | 83704 | 83485 | 3162 | 0.375 |  |  |  |  |  |  |  |
|  | 15 | 82124 | 82935 | 84289 | 84160 | 1191 | 0.540 |  |  |  |  |  |  |  |
|  | 35 | 85110 | 83188 | 82786 | 84393 | 1331 | 0.598 |  |  |  |  |  |  |  |
|  | 60 | 84482 | 85063 | 86893 | 85881 | 1130 | 0.489 |  |  |  |  |  |  |  |
|  | SEM | 2912 | 2470 | 958 | 1105 |  |  |  |  |  |  |  |  |  |
|  | *P*-value | 0.382 | 0.764 | 0.064 | 0.564 |  |  |  |  |  |  |  |  |  |
| Valid tags | 0 | 71460 | 64708b | 69862b | 67475 | 2959 | 0.430 | < 0.001 | 0.016 | 0.320 | 0.711 | 0.685 | 0.547 | 0.280 |
|  | 5 | 77042 | 72990ab | 76549ab | 74995 | 2594 | 0.692 |  |  |  |  |  |  |  |
|  | 15 | 75553 | 77688a | 77039ab | 72965 | 2307 | 0.504 |  |  |  |  |  |  |  |
|  | 35 | 76965 | 76379a | 75673ab | 74763 | 1922 | 0.864 |  |  |  |  |  |  |  |
|  | 60 | 79599 | 79983a | 79608a | 73889 | 1569 | 0.050 |  |  |  |  |  |  |  |
|  | SEM | 2069 | 2877 | 2141 | 2105 |  |  |  |  |  |  |  |  |  |
|  | *P*-value | 0.133 | 0.016 | 0.062 | 0.123 |  |  |  |  |  |  |  |  |  |
| Observed  OTUS | 0 | 144a | 92a | 115a | 128a | 20.7 | 0.384 | < 0.001 | < 0.001 | 0.922 | 0.231 | 0.351 | 0.115 | 0.006 |
|  | 5 | 73.0Ab | 10.8Bb | 43.3ABb | 15.5Bb | 10.9 | 0.006 |  |  |  |  |  |  |  |
|  | 15 | 74.8Ab | 14.0Cb | 42.0Bb | 28.0BCb | 6.10 | < 0.001 |  |  |  |  |  |  |  |
|  | 35 | 53.8b | 40.3b | 59.5b | 29.5b | 9.62 | 0.172 |  |  |  |  |  |  |  |
|  | 60 | 29.3Bb | 41.5Bb | 71.0Ab | 36.3Bb | 6.17 | 0.002 |  |  |  |  |  |  |  |
|  | SEM | 13.2 | 12.5 | 13.6 | 7.51 |  |  |  |  |  |  |  |  |  |
|  | *P*-value | < 0.001 | 0.003 | 0.011 | < 0.001 |  |  |  |  |  |  |  |  |  |
| Shannon | 0 | 4.12a | 4.29a | 4.37a | 3.70a | 0.519 | 0.798 | < 0.001 | < 0.001 | 0.133 | < 0.001 | < 0.001 | 0.237 | < 0.001 |
|  | 5 | 3.81Aa | 0.045Cb | 1.08Bb | 0.052Cb | 0.166 | < 0.001 |  |  |  |  |  |  |  |
|  | 15 | 3.17Aa | 0.040Cb | 1.30Bb | 0.266Cb | 0.251 | < 0.001 |  |  |  |  |  |  |  |
|  | 35 | 2.80Aa | 0.177Bb | 3.18Ab | 0.201Bb | 0.164 | < 0.001 |  |  |  |  |  |  |  |
|  | 60 | 1.10Bb | 0.172Cb | 3.24Ab | 0.290Cb | 0.216 | < 0.001 |  |  |  |  |  |  |  |
|  | SEM | 0.489 | 0.186 | 0.246 | 0.110 |  |  |  |  |  |  |  |  |  |
|  | *P*-value | 0.005 | < 0.001 | < 0.001 | < 0.001 |  |  |  |  |  |  |  |  |  |
| Simpson | 0 | 0.820a | 0.875a | 0.871a | 0.771a | 0.052 | 0.478 | < 0.001 | < 0.001 | 0.028 | < 0.001 | < 0.001 | 0.051 | < 0.001 |
|  | 5 | 0.865Aa | 0.008Cb | 0.264Bb | 0.008Cb | 0.018 | < 0.001 |  |  |  |  |  |  |  |
|  | 15 | 0.769Aa | 0.006Cb | 0.326Bb | 0.051Cb | 0.049 | < 0.001 |  |  |  |  |  |  |  |
|  | 35 | 0.734Ba | 0.030Cb | 0.809Aa | 0.036Cb | 0.023 | < 0.001 |  |  |  |  |  |  |  |
|  | 60 | 0.359Bb | 0.029Cb | 0.807Aa | 0.053Cb | 0.076 | < 0.001 |  |  |  |  |  |  |  |
|  | SEM | 0.083 | 0.019 | 0.043 | 0.015 |  |  |  |  |  |  |  |  |  |
|  | *P*-value | 0.005 | < 0.001 | < 0.001 | < 0.001 |  |  |  |  |  |  |  |  |  |
| Chao1 | 0 | 145a | 92.2a | 119a | 129a | 20.9 | 0.375 | < 0.001 | < 0.001 | 0.893 | 0.256 | 0.480 | 0.119 | 0.006 |
|  | 5 | 73.9Ab | 10.8Bb | 43.8ABb | 17.2Bb | 11.2 | 0.007 |  |  |  |  |  |  |  |
|  | 15 | 75.5Ab | 15.0Cb | 42.4Bb | 29.4BCb | 6.06 | < 0.001 |  |  |  |  |  |  |  |
|  | 35 | 55.1b | 42.0b | 62.2b | 30.6b | 10.4 | 0.201 |  |  |  |  |  |  |  |
|  | 60 | 33.2Bb | 46.1Bb | 71.3Ab | 38.0Bb | 5.98 | 0.003 |  |  |  |  |  |  |  |
|  | SEM | 13.2 | 12.6 | 14.2 | 7.85 |  |  |  |  |  |  |  |  |  |
|  | *P*-value | < 0.001 | 0.003 | 0.011 | < 0.001 |  |  |  |  |  |  |  |  |  |

CK, ensiling *L. chinensis* with 2.00 mL/kg fresh weight (FW) of distilled water; L, ensiling *L. chinensis* with 2.00 g/t FW of LAB inoculant and 2.00 mL/kg FW of distilled water; W, ensiling *L. chinensis* with 100 mL/kg FW of distilled water; LW, ensiling *L. chinensis* with 2.00 g/t FW of LAB inoculant and 100.0 mL/kg FW of distilled water. SEM, standard error of the mean. D, days after ensiling. Values with different uppercase letters (A, B, C, and D) indicate significant differences among treatments on the same day (*P* < 0.05). Values with different lowercase letters (a, b, ……, e) indicate significant differences among days after ensiling for the same treatment (*P* < 0.05)..
